# A systematic review of physical activity and sedentary behaviour research in the oil-producing countries of the Arabian Peninsula

**DOI:** 10.1186/s12889-016-3642-4

**Published:** 2016-09-21

**Authors:** Ruth Mabry, Mohammad Javad Koohsari, Fiona Bull, Neville Owen

**Affiliations:** 1 Office of the World Health Organization Representative, PO Box 476, Al Atheiba, Postal Code 130 Oman; 2 Behavioural Epidemiology Laboratory, Baker IDI Heart and Diabetes Institute, Melbourne, Australia; 3 Centre for Built Environment and Health, School of Population Health, the University of Western Australia, Perth, Australia; 4 Baker IDI & Heart Diabetes Institute, Swinburne University, Melbourne, Australia; 5 Faculty of Sport Sciences, Waseda University, Saitama, Japan; 6 Institute for Health and Ageing, Australian Catholic University, Melbourne, Australia; 7 Melbourne School of Population and Global Health, the University of Melbourne, Melbourne, Australia

**Keywords:** Physical activity, Sedentary behaviour, Arab

## Abstract

**Background:**

The dramatic rise in Noncommunicable Diseases (NCD) in the oil-producing countries of the Arabian Peninsula is driven in part by insufficient physical activity, one of the five main contributors to health risk in the region. The aim of this paper is to review the available evidence on physical activity and sedentary behaviour for this region. Based on the findings, we prioritize an agenda for research that could inform policy initiatives with regional relevance.

**Methods:**

We reviewed regional evidence on physical activity and sedentary behaviour to identify the needs for prevention and policy-related research. A literature search of peer-reviewed publications in the English language was conducted in May 2016 using PubMed, Web of Science and Google Scholar. 100 studies were identified and classified using the Behavioural Epidemiology Framework.

**Results:**

Review findings demonstrate that research relevant to NCD prevention is underdeveloped in the region. A majority of the studies were epidemiological in approach with few being large-scale population-based studies using standardised measures. Correlates demonstrated expected associations with health outcomes, low levels of physical activity (particularly among young people), high levels of sedentary behaviour (particularly among men and young people) and expected associations of known correlates (e.g. gender, age, education, time, self-motivation, social support, and access). Very few studies offered recommendations for translating research findings into practice.

**Conclusions:**

Further research on the determinants of physical activity and sedentary behaviour in the Arabian Peninsula using standard assessment tools is urgently needed. Priority research includes examining these behaviours across the four domains (household, work, transport and leisure). Intervention research focusing on the sectors of education, health and sports sectors is recommended. Furthermore, adapting and testing international examples to the local context would help identify culturally relevant policy and programmatic interventions for the region.

**Electronic supplementary material:**

The online version of this article (doi:10.1186/s12889-016-3642-4) contains supplementary material, which is available to authorized users.

## Background

Noncommunicable disease (NCD) accounts for a large portion of mortality and morbidity in the oil-producing countries of the Arabian Peninsula (Bahrain, Kuwait, Oman, Qatar, Saudi Arabia and United Arab Emirates; UAE) [1, 2]. A large majority of NCD mortality is due to cardiovascular disease. For example, more than a quarter of the adult population has high blood pressure and high blood glucose [3]. More than half of adults in the region are currently overweight or obese [3]. In the context of the links between maternal obesity and gestational diabetes and the health of unborn children globally [4], these regional prevalences combined with a high rate of congenital anomalies [2, 5] have far reaching implications for the health of future generations.

Insufficient physical activity (defined as less than 150 min of moderate physical activity per week) is one of the main contributors to health risk globally [3]. Sedentary behaviour (any waking behaviour characterized by an energy expenditure ≤1.5 METs while in a sitting or reclining posture [6, 7]) is a newer area of research; it has associations with all-cause and cardiovascular disease mortality, diabetes, and obesity when controlling for the influence of moderate-vigorous physical activity [8, 9]. The latest research indicates that a high level of moderate physical activity attenuates, but does not eliminate, the risk of high sitting time [10]. Despite the growing research confirming these associations, there is insufficient research documenting their relevance for the six oil-producing countries of the Arabian Peninsula.

The rapid socio-economic development of the region has contributed to a rise in urbanization, motorisation, trade liberalization and “western” dietary patterns [1, 11] which are widely recognized as key contributors to the rise of NCDs globally [12–14]. Although the radical changes in the food environment and consumption patterns in the region have been documented [1, 11], there is less evidence about the relationship between physical activity and sedentary behaviour and NCDs [15, 16]. There is also limited documentation of the shifts in occupational and transport (car-dependent) patterns in the region and their impact on physical activity [1]. Evidence that would help identify the social and cultural contexts that may limit people’s mobility, especially women, is sparse [15, 17]. The role of the hot arid climate has yet to be adequately specified [17, 18]. Thus, more thorough documentation of physical activity and sedentary behaviour in the region is an important priority, particularly as it relates to NCD prevention.

Research establishing patterns of physical activity and sedentary behaviour is well-documented in most other regions globally [14, 19–21]. The Behavioural Epidemiology Framework was developed as a simple approach to understand these patterns and build the evidence needed to inform public health action on physical activity and sedentary behaviour [22, 23]. It organizes research into the following phases:

- Phase 1. Identifying relationships of physical activity and sedentary behaviour with health outcomes
- Phase 2. Measuring physical activity and sedentary behaviour
- Phase 3. Characterizing prevalence and variations of physical activity and sedentary behaviour in populations
- Phase 4. Identifying the determinants of physical activity and sedentary behaviour
- Phase 5. Developing and testing interventions to influence physical activity and sedentary behaviour
- Phase 6. Using evidence to inform public health guidelines and policy

As a research framework, it helps identify research gaps and systemizes the development of a research agenda to inform and guide public health policy and practice. To be effective, regional evidence is needed to understand the contextual determinants of these behaviours and introduce regionally relevant policies to address them [1, 24]. We review the available evidence on physical activity and sedentary behaviour for the oil-producing countries of the Arabian Peninsula. Based on the findings, we prioritize an agenda for research that could inform policy initiatives in the region.

## Methods

### Search strategy

A literature search was conducted in May 2016 with PubMed, Web of Science and Google Scholar using the following search terms: active living; exercise; lifestyle; physical activity; walking; screen time; sedentary; sitting or television viewing; and the name of each country in the Region (Bahrain, Kuwait, Oman, Qatar, Saudi Arabia and United Arab Emirates) or Arab. The search was limited to peer-reviewed publications in the English language from any time period through April 2016. All articles were imported in an Endnote file to facilitate deduplication, screening and selection.

### Selection process

The initial search produced 3,560 articles, after deduplication. All articles were screened independently by two authors (RMM and MJK). Screening was conducted in two steps. In the first step, original English language articles on related disciplines published in peer-reviewed journals through April 2016 were included by judging from the title and source of articles. Publications in other languages, conference proceedings and theses as well as articles in unrelated disciplines were removed. By the end of this step, 347 articles remained.

In the second step, the abstracts and full texts were examined. The primary inclusion criteria were country specific studies which gathered original data, fit into any phase of the Behavioural Epidemiology framework [22, 23], and full-texts were available. Additional secondary inclusion criteria were used for the first three phases to facilitate within and cross-country comparison:

- Phase 1: Cross-sectional studies used a clearly described measure for physical activity/sedentary behaviour and prospective studies involved a physical activity intervention
- Phase 3: Studies clearly defined physical activity as meeting the recommendation of 150 min/week for adults or 60 min/day for children/adolescents.
- Phase 4: For demographic correlates, studies used a clearly described measure for physical activity/sedentary. The secondary inclusion criteria were not used for the studies examining the non-demographic articles to ensure a comprehensive review of available research in the region.

**Fig. 1:**
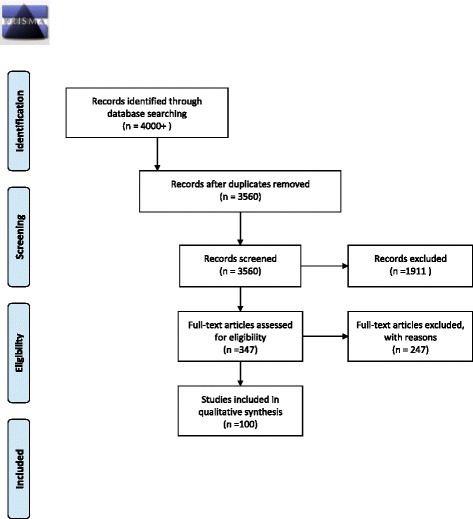
PRISMA 2009 Flow diagram

This resulted in a total of 100 articles. The flow diagram for article inclusion following PRISMA guidelines can be seen in Fig. 1 [25]. Since this was a systematic review of published research, rather than a study involving the collection of primary data, ethical clearance was not obtained.

### Data extraction, analysis and synthesis

Once the list of selected studies were identified, RMM extracted and MJK cross-checked the following for each: authors, country in which study was conduct, sample characteristics (number, size, gender, age), and physical activity/sedentary behaviour measurement tools. Key findings of each study were extracted and organized according to the Behavioural Epidemiology Framework. Differences in opinion in data extracted and placement within the framework were discussed to reach consensus. An ecological model that helps to classify potential multiple levels of influence on physical activity and sedentary behaviours-intrapersonal, social cultural, environmental [26, 27], was used to further organize the evidence for Phase 4 (the determinants of physical activity and sedentary behaviour).

### Risk of bias assessment

Two authors (RMM and MJK) independently assessed quality of studies included in the review. Studies were assessed for risk of bias using criteria adapted from the Cochrane risk of bias tool [28] and a tool for qualitative research studies [29]. All studies, except qualitative studies, were given a score of ‵1′ if they had an adequate description for each of five criteria: eligibility, randomization of participant selection or assignment for case–control studies, study sample (including number/size, age and gender), measurement of physical activity or sedentary behaviour appropriate for the aim of the study, and co-variates included in the data analysis. Qualitative studies were assessed for a clear description of eligibility, sample selection, study sample (including number/size, age and gender), utilization of standard definition for physical activity and/or sedentary behaviour, and analysis/interpretation.

## Results

Fourteen prospective studies and 29 cross-cross-sectional studies utilizing a clearly defined measure of physical activity and sedentary behaviour (such as the Global Physical Activity Questionnaire-GPAQ [30] or International Physical Activity Questionnaire-IPAQ [31]) were included in Phase 1, associations with health outcome (Table 1). Only 3 studies focused solely on measurement and adapted and/or validated a tool (Phase 2) [32–34]. Twenty-six population-based cross-sectional studies reporting on prevalence of these behaviours were included in Phase 3. Studies in Phase 4 included 10 population-based cross-sectional studies utilizing standard measures for physical activity and sedentary behaviour to examine the demographic risk factors influencing these behaviours as well as 25 studies examining a diversity of factors using various study methodologies. Only six studies that reported testing physical activity interventions were included in Phase 5 and none identified in Phase 6. Eleven studies were included in more than one phase (Additional file 1: Table S1).

**Table 1:** Number of studies on physical activity and sedentary behaviour in countries of the GCC according to phase of the behavioural epidemiology framework by population group

Phase of Behavioural Epidemiology Framework	Total Number of Studies								
	Physical Activity				Sedentary Behaviour				Total
	Adult	Adolescent	Children	Total	Adult	Adolescent	Children	Total	
1. Associations with Health Outcome	29	11	2	29	8	10	3	18	43
2. Measuring Behaviours		2	1	3		1		1	3
3. Prevalence and Variation	10	8		18	3	12	4	17	26
4. Correlates	25	9	1	35					35
5. Interventions	6			6					6
6. Policy	None								

Phases I and III studies and Phase IV studies for demographic correlates included population-based surveys that aimed to include a representative sample and used standard measures for PA; Two Phase II studies were regarding the same instrument; Phase IV studies for other correlates include all cross-sectional and qualitative studies on various supports and barriers to physical activity; Phase V studies included only those that described the intervention and reported on behaviour change due to the intervention

The review showed a relatively short history of research being conducted on physical activity in this region. All studies were published after the year 2000 with half (52) published in 2013 or later. Examining physical activity and/or sedentary behaviour was explicitly mentioned in the objectives of half (55) of the articles; the remaining focused more broadly on “risk factors” or “lifestyles”. Over half of the studies focused on populations in Saudi Arabia (57) and the UAE (16) with 8 or less articles about populations in each of the other countries of the region (Kuwait: 8; Oman: 7; Qatar: 6 and Bahrain: 5, not shown); the target populations were citizens of each country except for one study where the sample was South Asian immigrants [35].

A majority (86) focused on physical activity with only a few reporting on domain specific physical activity; work (2) [16, 36], transport (4) [16, 35–37], and/or leisure physical activity (5) [16, 37–39]. One-third (34) examined sedentary behaviour in the first 3 phases; all reported on TV viewing and/or computer time except for 8 studies reporting on total sitting time. Most studies focused on adults (62) and/or adolescents (28) with only 7 involving children less than 10 years. Very few studies (6) focused on translating knowledge into practice (Phases 5); these were only in adult populations. A majority (74) were cross-sectional descriptive epidemiology studies. The remaining were short-term (less than 1 year) intervention studies (18), long-term (6 or more years) prospective studies (2) [38, 40] or qualitative studies (6) focusing on physical activity.

### Findings of studies organized within the behavioural epidemiology framework

#### Phase 1 (Identifying relationships with health outcomes)

The review identified 14 prospective studies involving a physical activity intervention and 29 cross-sectional population-based studies utilizing a clear definition of physical activity to examine the association of physical activity with a health outcome (Table 2). Thirty studies sampled adult populations; the remaining studies were among young people, with only three including children under the age of 10 years [41–43]. The most common health outcome studied was obesity (21 studies) using BMI, waist circumference, waist-hip ratio and/or total body fat as the outcome measure. Most reported an inverse association of obesity with physical activity; one reported a positive association with total body fat [44] and two reported no significant association [41, 42]. The remaining studies identified associations with various clinical indicators like high blood pressure [35, 38, 45–49], diabetes [46, 50–52], bone health [37, 40, 53], the metabolic syndrome [16, 54], and Vitamin D deficiency [55, 56].

**Table 2:** Studies on the associations of physical activity and sedentary behaviour with health outcomes in oil-producing countries of the Arabian Peninsula (Behavioural epidemiology framework, Phase 1)

Lead author Country	Study design	Health Outcomes	Association	
			Physical Activity (type)	Sedentary Behaviour (type)
Prospective Studies				
Abdi S [50], United Arab Emirates	Diet and physical activity intervention (6 months)	HbA1c	−physical activity intervention)	
	35 adults aged 18–60 years with diabetes			
Al Saif A [45], Saudi Arabia	Aerobic and anaerobic intervention (3 months)	BMI	−(Aerobic intervention)	
		Blood Pressure		
	40 obese adults aged 18–25 years	Heart Rate		
		Maximum oxygen consumption	+ (Aerobic intervention)	
		Maximum voluntary ventilation	+ (Aerobic and anaerobic interventions)	
Al-Eisa E [148], Saudi Arabia	Exercise intervention (8 weeks)	Serum cotinine	−(Exercise intervention for both smokers and non-smokers)	
	150 men aged 18–55 years	Serum cortisol		
		Testosterone		
		Mood and physical symptoms scale	−(Exercise intervention for smokers)	
		Free radicals	+ (Exercise intervention for both smokers and non-smokers)	
Al-Eisa E [64], Saudi Arabia	Exercise intervention (3 weeks)	Insomnia	−(exercise intervention)	
	76 women university students aged 19–25 years	Depression		
		Attention span	+ (Exercise intervention)	
Alghadir AH [53], Saudi Arabia	Exercise intervention (12 weeks)	BMI	−(Exercise intervention)	
	100 adults aged 30–60 years	Waist-Hip ratio		
		Serum levels of Copper, Zinc and bone-specific alkaline phosphatase		
		Osteoporosis T-score		
		Bone mineral density	+ (Exercise intervention)	
		Serum levels of Calcium and Manganese		
Alghadir AH [149], Saudi Arabia	Exercise intervention (4 weeks)	Salivary cortisol, lactate and testosterone levels	+ (Exercise intervention)	
	16 men students aged 15–25 years			
Al-Ghimlas F [46], Kuwait	Exercise intervention (12 weeks)	Weight	−(Exercise intervention)	
	58 adults aged 15+ years	BMI		
		Waist and hip circumferences		
		Diastolic blood pressure		
		Resting Heart Rate		
		HbA1c		
		LDL cholesterol		
		Body fat composition		
		Peak oxygen uptake Muscular strength	+ (exercise intervention)	
Ardawi MM [37], Saudi Arabia	Exercise intervention (8 weeks)	Serum bone-formation markers:	−(exercise intervention)	
	160 women aged 20–49 years			
		Sclerostin		
		CTX		
		IGF-I	+ (exercise intervention)	
		OC		
		PINP	N (exercise intervention)	
		bone-ALP		
		PTH		
		NTX		
Kneffel Z [47], Qatar	Exercise intervention (10 weeks)	Weight	−(Exercise intervention)	
	36 students aged 18–30 years	BMI		
		Body fat composition		
		Diastolic blood pressure		
Rouzi AA [40], Saudi Arabia	Prospective cohort (6 years)	All fragility related fractures	−(Total PA)	
	707 healthy post-menopausal women aged 50+ years			
Sadiya A [51], United Arab Emirates	Lifestyle intervention including physical activity (12 weeks)	Weight	−(Lifestyle intervention)	
		BMI		
	45 obese or obese with type 2 diabetes adults aged 18–50 years	Body fat composition		
		WC		
		Fasting blood glucose	−(Lifestyle intervention for obese with diabetes)	
		HbA1c		
Salman RA [38], Saudi Arabia	Exercise intervention (11–year)	Hypertension	−(leisure PA)	
	916 normotensive adults with diabetes aged 20+ years			
Tomar RH [52], Saudi Arabia	Exercise intervention (12 weeks)	Glycemic control	+ (exercise intervention)	
	24 adult men with type 2 diabetes aged 25–55			
Cross-sectional: Adults				
Al-Daghri NM [150], Saudi Arabia	Cross-sectional	Irisin levels	+ (Total PA for healthy adults	
	164 adults aged 30–75 years			
			N (total PA for adults with diabetes)	
Al-Hamdan NA [48], Saudi Arabia	Cross-sectional	Hypertension	−(Work, transport and leisure PA)	
	4758 adults aged 15–64 years			
Al-Mahroos F [36], Bahrain	Cross-sectional	BMI	−(Occupational activity)	−(TV time)
	2013 adults; men aged 40–59 years and women aged 50–69 years		−(Walking and cycling, significant in only men)	
Almajwal MA [91] ^4^, Saudi Arabia	Cross-sectional	BMI	−(Total PA)	
	362 Non-Saudi hospital nurses			
Al-Nozha MM [39] ^3, 4^, Saudi Arabia	Cross-sectional	BMI	−(Leisure PA)	
	17,395 adults aged 30–70 years	WC	−(Leisure PA)	
Al-Thani [151], Qatar	Cross-sectional	BMI	N (Total PA)	
	2496 adults aged 18–64 years	WC	−(Total PA)	
Ardawi MM [37], Saudi Arabia	Cross sectional	Serum bone-formation markers:	−(Walking or exercising)	
	1235 women aged 20–49 years			
		Sclerostin		
		FSH		
		CTX		
		IGF-I	+ (Walking or exercising)	
		OC		
		PINP		
		bone-ALP		
		PTH		
		E2		
		NTX		
Al-Kilani H [44], Oman	Cross-sectional	Total body fat	+ (exercise and physical activity scores)	
	202 school students aged 18–25 years			
Basulaiman M [67], Saudi Arabia	Cross-sectional	Hypercholesterolemia	N (Total PA)	N (Total TV/computer time)
	10735 adults aged 15+ years			
		Borderline Hypercholesterolemia	N (Total PA)	+ (Total TV/computer time)
El-Aty MA [54] ^3^, Oman	Cross-sectional	Metabolic Syndrome	N (Total PA)	+ (Total sitting time)
	3137 adults aged 18+ years			
El Bcheraoui C [49], Saudi Arabia	Cross-sectional	Hypertension	N (Total PA)	N (Total TV/computer time)
	10735 adults aged 15+ years			
		Borderline hypertension	+ (Total PA−moderate active only)	N (Total TV/computer time)
El-Ghazali S [152], Kuwait	Cross-sectional	BMI	−(Total PA)	
	320 college students, 17–26 years			
Hegazy AM [153], Saudi Arabia	Cross-sectional study	Lower back pain	−(Total PA)	+ Prolonged sitting
	174 women, half with lower back pain for 3+ months (20–45 years)			
Mabry RM [16], Oman	Cross-sectional	The Metabolic Syndrome	−(Work PA)	+ (total sedentary behavior but not significant after further adjustment for PA)
	1,335 adults age 20 years and older		−(Transport PA)	
			N (Leisure PA)	
Memish ZA [154], Saudi Arabia	Cross-sectional	BMI	−(Total PA, men only)	
	10,735 adults 15+ years			
Moradi-Ladek M [68], Saudi Arabia	Cross-sectional	Self-rated health		+ (TV/computer time)
	10,735 adults 15+ years			
Shah SM [35], United Arab Emirates	Cross-sectional	Hypertension	−(Walking 30+ mins/day)	
	1,375 South Asian immigrants men 18+ years			
Tuffaha M [55], Saudi Arabia	Cross-sectional	Vit. D deficiency	N (Total PA)	
	10,735 adults 15+ years			
Prospective: Children and adolescents				
Al Saweer A [155], Bahrain	Behavioural intervention including physical activity	Weight	−(Behavioural intervention)	
		BMI		
	13 obese adolescents			
Cross-Sectional: children and adolescents				
Al Junaibi A [41], UAE	Cross-sectional	BMI	N (time spent walking, moderate activity, vigorous activity)	−(computer time in girls)
	1,541 students aged 6–19 years			
				+ (computer time in boys)
				N (time spent sitting)
Al-Haifi AA [156], Kuwait	Cross-sectional	Sleep duration	N (Moderate and/or vigorous activity)	−(TV and computer time in boys)
	906 school students aged 14–19 years			
Al-Haifi AR [60], Kuwait	Cross-sectional	BMI	−(Moderate and Vigorous PA)	N (TV and Computer time)
	906 school students aged 14–19 years	WC		
			−(Vigorous PA)	
Al-Hazzaa HM [61], Saudi Arabia	Cross-sectional	BMI	−(vigorous PA)	N (TV/screen time)
	2,906 school students aged 14–19 years		N (Total PA)	
		WtHR	−(vigorous PA)	
			N (Total PA)	
Al-Hazzaa HM [157], Saudi Arabia	Cross-sectional	Sleep duration	+ (Total PA)	+ (screen time)
	2,868 secondary-school students aged 15–19 years			
Al-Kilani H [44], Oman	Cross-sectional	Total body fat	+ (exercise and physical activity scores)	
	202 school students aged 18–25 years			
Al-Nakeeb Y [57] ^3^, Saudi Arabia	Cross-sectional	BMI	−(Total PA and walking)	+ (computer use and total TV and computer time)
	1,138 school students aged 15–17 years			
Al-Nuaim AA [58] ^3^, Saudi Arabia	Cross-sectional	BMI	−(Total PA)	+ (sitting time)
	1,270 school students aged 15–19 years	WC	−(Total PA)	+ (sitting time)
Alqahtani N [62], Saudi Arabia	Cross-sectional	BMI	−(Total PA, boys only)	−(Screen time)
	370 school children aged 14–19 years			
Alrashidi M [59], Kuwait	Cross-section	BMI		+ (TV time in boys)
	635 children aged 11–14 years			
Kerkadi A [42], UAE	Cross-sectional	BMI	N (Total PA)	+ (TV time)
	900 female primary school children aged 5–14 years			
Muhairi SJ [56], UAE	Cross-sectional	Vitamin D levels	+ (Total PA)	
	315 healthy adolescents aged 15–18 years			
Yousef S [43] ^3^, UAE	Cross-sectional	Childhood behavioral problems		+ (TV and Video games time)
	197 school children aged 5–15 years			

*BMI* body mass index, *PA* physical activity, *ST* sitting time, *WC* waist circumference, *WtHr* waist to hip ratio, + positive association,−inverse association N non-significant, Super-script number (s)−cross referencing to additional table (s) article in which article is included

Twenty-one articles examined associations of sedentary behaviour with a health outcome. Associations of obesity with sedentary behaviour were less conclusive than with physical activity: four studies reported a positive association with different types of sedentary behaviour [42, 57–59], two found no association [60, 61], two reported an inverse association [36, 62] and one reported a positive association with computer time among boys but an inverse association among girls [41]. Another key health outcome studied was the metabolic syndrome; two found a positive association with total sitting time [16, 54] but one reported that this association did not remain after further adjustment for physical activity [16].

#### Phase 2 (Measuring physical activity and sedentary behavior)

Three studies tested the validity and reliability of Arabic physical activity questionnaires. Two measured physical activity behaviour: the Arabic version of the Questionnaire l’ Activite Physique en Altitude Chez les Enfants for children under the age of 10 years [34] and the tool for the Arab Teens Lifestyle Study (ATLS) [33]. The third tested the psychometric properties of the Arabic Version of the Physical Activity Self-Efficacy Scale for Adolescents [32]. All were identified as valid physical activity instruments for young people in the Arab world.

Across all remaining studies the definitions and measures used for both physical activity and sedentary behaviour varied widely across studies. Several different tools were used to measure physical activity and sedentary behaviour (Additional file 1: Table S1). Self-report was the most common form of reporting (73). Only seven studies used objective measures for physical activity (pedometers and accelerometers); four studies used them as the only measure of behaviour [63–66]. None used objective measures for sedentary behaviour.

Most studies (75) examined self-report derived measures of total physical activity. TV/computer use and/or screen time was the most common proxy measure for sedentary behaviour (32); only six studies reported total sitting time. Some studies used reliable and validated instruments like the IPAQ/IPAQ-short (physical activity-12 studies; sedentary behaviour-5 studies [41, 49, 56, 67, 68]), GPAQ (physical activity-11 studies, sedentary behaviour-4 studies [16, 54, 69, 70]) and the ATLS (physical activity-16 studies, sedentary behaviour-15 studies). Many studies developed their own tools (29 studies examining physical activity and 8 studying sedentary behaviour), eleven provided very limited description of the tools in terms of measuring frequency, intensity and duration of physical activity. Seven studies developed/adapted tools for gathering cognitive and psychosocial data relevant to physical activity such as perceived barriers [71–75], stages of change [73, 75] and self-efficacy [73, 75]. None of the studies in this review used tools concerning the physical environment and physical activity.

#### Phase 3 (Characterizing Prevalence and variation in populations)

Of the 27 studies reporting on the prevalence of physical activity (Table 3), 11 were on the adult population in four countries: Oman, Qatar, Saudi Arabia and the United Arab Emirates. Four were population-based with three gathering data nationally through household interviews [39, 54, 69] and one by telephone in one city [76]. Two studies were conducted in Saudi Arabia with large differences in the prevalence of physical activity; for a national study the prevalence was very low (Men: 6.1 %, Women: 1.9 %) [39] while in the telephone-based study in Riyadh reported that more than half were met physical activity recommendations (Men: 56.3 %; Women: 65.7 %) [76]. The national study in Oman reported higher prevalence rates than the national household study in Saudi Arabia and Qatar (Women: 44.2 %) with similar rates as in Riyadh (Men: 68.0 %, Women: 59.5 %) [54].

**Table 3:** Prevalence of physical activity and sedentary behaviour in oil-producing countries of the Arabian Peninsula (Behavioural epidemiology framework, Phase 3)

Author Country	Sample	Physical Activity and Sedentary Behaviour Measurement Tool	Physical Activity	Sedentary Behaviour
Adults				
Al-Hazzaa HM [76] ^4^, Saudi Arabia	1,064 adults aged 15–78 years	IPAQ short	Men: 56.3 %	
			Women: 65.7 %	
			Total: 59.4 %	
Allam AR [158], Saudi Arabia	194 medical students	IPAQ short	Men: 36.2 %	
			Women: 35.0 %	
			Total: 35.5 %	
Al-Nozha MM [39] ^2, 4^, Saudi Arabia	17,395 adults aged 30–70 years	Validated questionnaire on Leisure time physical activity and walking	Men: 6.1 %	
			Women: 1.9 %	
Al Thani M [69], Qatar	747 women aged 18–64 years	GPAQ	Women: 44.2 %	Mean total sitting time:
				183.6 ± 168.3 min/day
Awadalla NJ [72] ^4^, Saudi Arabia	1257 health professional college students	IPAQ short	Men: 43.7 %	
			Women: 41.2 %	
			Total: 42.0 %	
Banday AH [159], Saudi Arabia	106 Primary Health care Physicians aged 27–63 years	GPAQ	Total: 65.2 %	
Carter AO [160], UAE	175 Medical students aged 19–27 years	Nurses’ Health Study II	Total: 67.0 %	
El-Aty, MA [54] ^2^, Oman	3137 adults aged 18+ years	GPAQ	Men: 68.0 %	Prevalence (6+ hrs/day):
			Women: 59.5 %	Men: 21.5 %
			Total: 63.4 %	Women: 25.6 %
				Total: 23.7 %
Khalaf A [161], Saudi Arabia	663 female university students	ATLS	Women: 62.4 %	
Koura MR [162], Saudi Arabia	370 women college students	GPAQ	Women: 46.8 %	
Mabry RM [70] ^4^, Oman	1,335 adults aged 20 years and older	GPAQ		Prevalence (3+ hrs/day):
				Men: 64.8 %
				Women: 37.8 %
				Total: 45.3 %
Children and adolescents				
Al-Hazzaa HM [86] ^4^, Saudi Arabia	2,866 school students aged 15–19 years	ATLS	Boys: 43.8 %	
			Girls: 20.2 %	
			Total: 31.5 %	
Al-Hazzaa HM [78], Saudi Arabia	2,908 secondary-school students aged 14–19 years	ATLS	Boys: 55.5 %	Computer/TV time >2 h/day:
			Girls: 21.9 %	Boys: 84.0 %
				Girls: 91.2 %
Al-Hazzaa HM [81], Saudi Arabia	2,886 students aged 15–19 years	ATLS	Boys: 55.0 %	Computer/TV time >3 h/day:
			Girls: 21.7 %	Boys: 69.8 %
				Girls: 81.8 %
Al-Hazzaa HM [80] ^4^, Saudi Arabia	1,648 students aged 14–18 years	ATLS	Boys: 53.4 %	Mean computer/TV time (hrs/day):
			Girls: 19.1 %	
			Total: 36.0 %	Boys: 5.31 ± 3.1
				Girls: 5.89 ± 3.3
				Computer/TV time >2 h/day:
				Boys: 84.2 %
				Girls: 91.6 %
				Total: 88.0 %
Al-Hazzaa HM [65], Saudi Arabia	224 preschool children aged 3.4 to 6.4 years	unknown		Mean TV time (minutes/day):
				Boys: 162.4 ± 69.9
				Girls: 147.7 ± 61.7
				Total: 154.8 ± 66.1
Allafi A [77], Kuwait	906 Adolescents aged 14–19 years	ATLS	Boys:70.5 %	% watch >2 h of TV/day:
			Girls: 39.2 %	Boys: 69.7 %
				Girls: 72.7 %
				% use computers >2 h/day:
				Boys: 62.1 %
				Girls: 70.0 %
Al-Nakeeb Y [57]^2^, Saudi Arabia	2,290 school students aged 15–17 years	ATLS	Boys: 45.8 %	Mean time watching TV (hrs/day):
			Girls: 4.5 %	
			Total: 26.0 %	Boys: 2.51
				Girls: 2.61
				Mean computer time (hrs/day):
				Boys: 2.41
				Girls: 3.18
Al-Nuaim AA [58] ^2^, Saudi Arabia	1,270 school students aged 15–19 years	ATLS	Boys: 44.5 %	Mean time watching TV (hrs/day):
			Girls: 4.0 %	
				Boys: 2.49
				Girls: 2.60
				Mean computer time (hrs/day):
				Boys: 2.43
				Girls: 3.19
Farghaly NF [163], Saudi Arabia	767 students aged 7–20 years	unknown		Mean TV time (hrs/day)
				Total: 1.0 ± 1.0
				Mean computer game time (hrs/day)
				Total: 0.7 ± 0.9
Gharib NM [164], Bahrain	2,594 school children aged 6–18 years	Unknown		Mean hours of TV/Video/week:
				Boys: 11.5
				Girls: 31.2
				Mean hours of computer time/week:
				Boys: 3.3
				Girls: 2.7
Kilani H [82], Oman	802 adolescents aged 15–18 years	ATLS	Boys: 66.7 %	Mean screen time (hrs/day):
			Girls: 23.1 %	Boys: mean 2.86 SD2.3
				Girls: mean 3.70 SD2.9
Mahfouz AA [84], Saudi Arabia	1,869 adolescent aged 11–19 years	CDC Adolescent Health adapted		Watched > 3 h TV/daily:
				Boys: 38.0 %
				Girls: 52.7 %
Mahfouz AA [83], Saudi Arabia	2,696 adolescent school boys aged 11–19 years	Arabic version of CDC Adolescent Health Survey		Watched > 3 h TV/daily:
				Total: 38 %
Musaiger AO [85], Saudi Arabia	512 girl school students aged 12–19 years	unknown		≥3 h TV time/day:
				Girls: 60.9 %
Yousef, S [43] ^2^, UAE	197 school children aged 6–10 years	Unknown		% TV viewing/Video games > 2 h/day:
				Total: 62.9 %
Youssef RM [74], Oman	439 secondary-school students aged 15–20 years	unknown		% TV time ≥3 h/day:
				Boys: 21.1 %
				Girls: 25.3 %
				Total: 23.2 %
				% computer ≥3 h/day:
				Boys: 26.6 %
				Girls: 31.5 %
				Total: 29.2 %

*GPAQ* global physical activity questionnaire, *IPAQ* international physical activity questionnaire, *ATLS* Arab teens lifestyle student questionnaire

Physical activity presented as percentage meeting recommendations: 150 min of moderate-intensity per week for adults and 60 min of moderate-intensity 7 days a week for adolescents except for Qatar which is for at least 5 days a week; Sedentary Behaviour presented as prevalence (%) or Mean sitting time; All studies were population-based surveys that aimed to include a representative sample and used standard measures for PA

Super-script number (s)−cross referencing to additional table (s) article in which article is included

Sixteen country-specific studies from all six countries except Qatar reported on the prevalence of physical activity in the adolescent population. Eight school-based studies utilized the ATLS; among them, the lowest and highest reported prevalence of physical activity (doing at least 60 min moderate physical activity on 7 days a week) were 43.8 to 70.5 % for boys and 4 to 39.2 % for girls in Saudi Arabia and Kuwait respectively [57, 58, 77–82]. Prevalence rates across all studies showed consistently higher rates among boys than girls.

Sedentary behaviour was reported across 18 studies. Only three were national surveys among adult populations in Oman and Qatar; each study presented their data differently. Two studies were secondary analyses of the same survey conducted in Oman; one reported that a quarter of adults (23.7 %) sat 6 or more hours/day [54] and the other reported that nearly half (45.3 %) sat for at least 3 h/day [70]. The third study reported that the mean total sitting time was 183.6 min/day (SD: 168.3 min/day) for women in Qatar [69].

All 15 studies conducted with child and adolescents reported on computer, TV and/or total screen time. Like the adult studies, data were presented differently: mean TV and/or computer time or a prevalence of computer and/or TV of greater than 2 or 3 hours per day. Two studies reported mean computer/TV times with higher rates in girls (Oman: 3.70 ± 2.9 h/day; Saudi Arabia: 5.89 ± 3.3 h/day) than boys (Oman: 2.86 ± 2.3 h/day; Saudi Arabia 5.31 ± 3.1 h/day) [80, 82]. Four studies from Saudi [83–85] and Oman [74] reported on the prevalence of watching 3 or more hours of TV; the highest percentage were girls (60.9 %) in Jeddah, Saudi Arabia [85] and boys (38.0 %) in Abha, Saudi Arabia [84]. These rates were higher than seen in Oman; girls: 25.3 % and boys: 21.1 % [74]. The only study among preschool students reported a mean TV time of 154.8 ± 66.1 min/day [65].

### Phase 4 (Identifying the determinants)

Thirty-five studies examined the correlates of physical activity (Table 4). Population-based surveys conducted in Oman and Saudi Arabia consistently reported that gender and education were associated with physical activity with men being more physically active than women [39, 76, 86–90] and people with lower education were more active than their more educated counterpart [70, 76, 90, 91]. All studies reported an association of physical activity with age with younger people being more active than older people [39, 70, 76, 87, 89, 90], except for Oman, where one study reported a direct correlation of age and physical activity among men [70].

**Table 4:** Factors associated with physical activity in oil-producing countries of the Arabian Peninsula (Behavioural epidemiology framework, Phase 4)

Correlate	Association	References
Demographic		
Age	−	Al-Hazzaa, 2007 [76] ^3^; Al-Hazzaa HM [80] ^3^; Al-Nozha, 2007 [39] ^2, 3^; Al-Sobayel, 2015 [87]; Amin, 2012 [89]; Amin, 2011 [90]; Mabry, 2012 [70] (women only) ^3^
	+	Mabry, 2012 [70] (men only)
Gender (male)	+	Al-Hazzaa, 2007 [76]; Al-Hazzaa, 2014 [86] ^3^; Al-Nozha, 2007 [39]; Al-Sobayel, 2015 [87]; Amin, 2012 [89]; Amin, 2011 [90]; Duncan, 2015 [88]
Education	−	Al-Hazzaa, 2007 [76]; Almajwal, 2015 [91] ^2^; Amin, 2011 [90]; Mabry, 2012 [70] (men only)
Marital Status (married)	+	Almajwal, 2015 [91]; Al-Nozha, 2007 [39]; Mabry, 2012 [70] (men only)
	−	Khalaf, 2013 [161] (women only)
Employment (employed)	+	Mabry, 2012 [70]
Intrapersonal		
Lack of time	−	Al-Hazzaa, 2014 [86]; Al-Rafaee, 2001 [118]; Al-Otaibi, 2013 [75]; Ali, 2010 (Q) [18]; Ali, 2008 (Q) [119]; Alsubaie, 2015 [94]; Awadalla, 2014 [72] ^3^; Berger, 2009 (Q) [120]; Daradkeh, 2015 [95]; Hashim, 2013 [121]; Gawwad, 2008 [73]; Mabry, 2013 (Q) [17]; Musaiger, 2014 [122]; Serour, 2007 [123]; Youssef [74]
Self-motivation	−	Al-Rafaee, 2001 [118]; AlQuaiz, 2009 [71]; Alsubaie, 2015 [94]; Awadalla, 2014 [72]; Berger, 2009 (Q) [120]; Mabry, 2013 (Q) [17]; Youssef [74]
Perceived health	−	AboZaid, 2010 [165]; Al-Rafaee, 2001 [118]; Alsubaie, 2015 [94]; Awadalla, 2014 [72]; Serour, 2007 [123]
Limited knowledge/awareness	−	Ali, 2008 (Q) [119]; Daradkeh, 2015 [95]; Taha, 2008 [166]
Consumption of fruits	+	Al-Hazzaa, 2014 [86]; Al-Kahtani, 2015 [66]; Al-Sobayel, 2015 [87]; Duncan, 2015 [88]
Consumption of foods high in fats/salt/sugar	+	Al-Hazzaa, 2014 [86]; Al-Sobayel, 2015 [87]; Duncan, 2015 [88]; Faris, 2015 [167]
Knowledge PA is important	+	Donnelly, 2012 (Q) [92]; Sulaiman, 2009 (Q) [93]
Perceived skills/fitness	−	Awadalla, 2014 [72]; Gawwad, 2008 [73]
Enhance appearance/muscles	+	Alsubaie, 2015 [94]; Daradkeh, 2015 [95]
Consumption of milk	+	Al-Hazzaa, 2014 [86]; Al-Sobayel, 2015 [87]
Consumption of vegetables	+	Al-Hazzaa, 2014 [86]; Al-Sobayel, 2015 [87]
Belief in Overweight as normal	−	Ali, 2008 (Q) [119]
Attitude to changing diet	−	Ali, 2008 (Q) [119]
Self efficacy	+	Al-Eisa, 2012 [63]
Locus of control	−	Al-Otaibi, 2013 [75]
Stage of change	+	Al-Otaibi, 2013 [75]
Fear of criticism	−	AboZaid, 2010 [165]
Maintain health	+	Daradkeh, 2015 [95]
Shift duty	−	Almajwal, 2015 [91]
Social and cultural		
Norms limiting women’s mobility	−	Amin, 2010 [90]; Berger, 2009 (Q) [120]; Mabry, 2013 (Q) [17]; Sulaiman, 2009 (Q) [93]
Norms prioritizing women’s care-taking role/limiting self-care role	−	Donnelly, 2012 (Q) [92]; Sulaiman, 2009 (Q) [93]
Social support	−	Al-Otaibi, 2013 [75]; AlQuaiz, 2009 [71]; Alsubaie, 2015 [94]; Awadalla, 2014 [72]; Amin, 2010 [90]; Donnelly, 2012 (Q) [92]
Low value of PA	−	Ali, 2008 (Q) [119]; Awadalla, 2014 [72]; Mabry, 2013 (Q) [17]
Norms promoting overeating	−	Ali, 2008 (Q) [119]
Lack of role models		Berger, 2009 (Q) [120]
Physical Environment		
Availability of physical activity facilities	−	Al-Rafaee, 2001 [118]; Alsubaie, 2015 [94]; Awadalla, 2014 [72]; Ali, 2010 (Q) [18]; Ali, 2008 (Q) [119]; AlQuaiz, 2009 [71]; Amin, 2010 [90]; Donnelly, 2012 (Q) [92]; Mabry, 2013 (Q) [17]
Weather	−	Ali, 2008 (Q) [119]; Ali, 2010 (Q) [18]; Amin, 2010 [90]; Berger, 2009 (Q) [120] Mabry, 2013 (Q) [17]; Musaiger, 2014 [122]; Sulaiman, 2009 (Q) [93]
Safety	−	Ali, 2008 (Q) [119]
Transportation	+	Al-Kahtani, 2015 [66];
Population Policy Level		
Ineffective health communication	−	Ali, 2008 (Q) [119]; Mabry, 2013 (Q) [17]
Limited resources (general) allocated for physical activity promotion	−	Mabry, 2013 (Q) [17]
Ineffective PA-supportive policies in colleges	−	Berger, 2009 (Q) [120]
Individual-based Policy Level		
Lack of Time	−	Al-Doghether, 2007 [127]; Al-Ghawi, 2009 [128]
Health personnel limited knowledge/awareness of benefits of PA	−	Al-Doghether, 2007 [127]
Limited material resources in health centres (teaching materials, guidelines)	−	Al-Ghawi, 2009 [128]; Ali, 2008 (Q) [119]
Lack of specialty clinics at primary health care level	−	Al-Ghawi, 2009 [128]
Limited availability of human resources (i.e., dietitians)	−	Ali, 2008 (Q) [119]

Studies for the demographic correlates included population-based surveys that aimed to include a representative sample and used standard measures for physical activity; all other studies were cross-sectional studies using various methodologies except those marked Q to denote qualitative studies

Super-script number (s)−cross referencing to additional table (s) article in which article is included

In addition to the demographic correlates, the studies explored other factors associated with participation in physical activity, including intrapersonal, social/cultural, physical environment and policy level correlates. The most frequently identified barriers (negative association) of physical activity identified included: time, self-motivation, perceived health, norms limiting women’s mobility or prioritizing her care-taking role, social support, availability of facilities, limited capacity within health institutions and weather. Positive support for participation in physical activity mentioned in more than one study was the knowledge that physical activity is important [92, 93] and desire to enhance one’s appearance [94, 95]. Healthy diet, such as the consumption of fruits and vegetables, were also reported as positive correlates several studies [66, 86–88].

Only one study examined the correlates of sedentary behaviour [70]. It reported that younger women have higher sitting times than older women and more-educated men have higher sitting times than those less educated. Other correlates reported included employment status, smoking and obesity.

### Phase 5 (Developing and testing interventions)

Only six studies reported on interventions conducted in Bahrain, Saudi Arabia and UAE; three reported increases in physical activity [96–98] (Table 5). The duration for all studies was less than one year. Four were 6 months or less [96, 97, 99, 100]. Only one study included a post-intervention follow-up [99]. The target group varied with three involving university students [96, 97, 101], two addressing adults with chronic disease [99, 100] and only one targeting the general adult population [98]. Two supplemented awareness raising activities; one involved Instagram motivational messages to encourage the use of an exercise workout video [96] and the other encouraged participants to keep a 1 week log on physical activity [97]. The former was the only one of the six studies that followed a case–control methodology.

**Table 5:** Physical activity related interventions in oil-producing countries in the Arabian Peninsula (Behavioural epidemiology framework, Phase 5)

Lead Author Country	Target Group (Size)	Description of Intervention	Results
Abduelkarem A [99], UAE	Adults with type two diabetes visiting community pharmacies, aged 28–75 years (59)	3-month intervention where community pharmacies dispense self-care reminders (including physical activity advise); assessment carried out at 3-month, 6-month and 24 month by interview survey (59 % response rate)	Significant increases in physical activity during intervention period; but not at 6 and 24-months
Al-Eisa E [96], Saudi Arabia	Women university students aged 18–25 years (58)	4-week intervention involving Instagram educational and motivational messages to exercise using a 37 min of cardio work out video (81 % response rate)	Significant difference between case and controls to adherence to programme; 17 % cases compared to 4 % controls exercised 8+ times during intervention period
Barss P [97], UAE	1st year medical students (41)	Lifestyle curriculum involving 5 lectures; one family lifestyle history assessment; 1 week log on dietary intake and physical activity and an oral presentation	Significant number of students reported increase in physical activity and stair usage following completion of lifestyle course including 46 % starting exercising regularly and 63 % started using stairs more frequently
Grant N [101], Bahrain	Families (30)	8-month family studies programme for 3rd year medical students conducted 10–12 family visits over a period of 8 months.	Two-thirds of families reported in qualitative interviews positive behaviour changes including increase in levels of physical activity
Midhet FM [98], Saudi Arabia	Community (population size not reported)	1 year intervention in all PHC centres involving training physicians on lifestyle counselling and regular health center-based lectures conducted by health educators and medical students	A pre-post test community-based survey found significant increase in levels of brisk walking in men after adjustment for key demographic variables
Sharaf F [100], Saudi Arabia	Adults with hypertension, diabetes and coronary artery disease visiting PHC clinics (population size not reported)	6-month intervention involving training of PHC physicians and health educators aimed to increase knowledge and skills on patient education with a focus on diet, smoking and physical activity	No significant change in physical activity

All studies provided a brief description of the intervention and reported on behavior change due to the intervention

### Risk of Bias

The two reviewers independently reviewed the methodological quality of the studies included in the review. Quality ratings ranged between 1 and 5; discrepancies in ratings were discussed to reach agreement (Additional file 1: Table S1). For the quantitative studies (*n* = 94), the mean score was 3.6 with only a quarter (25.5 %) rated 5, the highest score (Additional file 1: Table S1). A vast majority (90 studies or 95.7 %) provided an adequate description of the study sample, 78 studies (83.0 %) provided an adequate description of random sampling, 62 studies (66.0 %) provided a clear description of the eligibility criteria, 61 studies (64.9 %) utilized a valid measure of physical activity and/or sedentary behaviour and 45 studies (47.9 %) adjusted for co-variates. For the six qualitative studies, the mean score was 4.2. All studies provided an adequate description of the eligibility criteria, study sample and the analysis and interpretation and five (83.3 %) provided an adequate description of the sample selection process. However, only two (33.3 %) adhered to the broad definition of physical activity vs the terminology of “exercise” or “sport.” When looking at the studies according to the five phases, the mean score was markedly lower for Phase 5 (2.3) and Phase 2 (3.0) studies which had fewer number of studies compared to those assigned to the other phases (Phase 1: 3.9, Phase 2: 3.7 and Phase 4: 3.6).

## Discussion

The findings of this review have identified relevant evidence and some of the limitations in understanding physical activity, sedentary behaviours and public health, an emerging area of knowledge in the Arabian Peninsula. Although 100 publications were identified since 2000, over half of these were published since 2013. This research was spread unevenly across the behavioural epidemiology phases used to structure our review of the evidence [22, 23]. The majority of published studies focussed on assessing health outcomes (Phase 1 *n* = 43), prevalence (phase 3, *n* = 26) or identifying the correlates of physical activity and sedentary behaviours (phase 4 *n* = 35).

Far fewer published studies addressed the measurement of physical activity and sedentary behaviours (phase 2, *n* = 3) [32–34] or the testing of interventions (phase 5, *n* = 6). Publications were found from all six countries in the study area, although were mostly focused on adults rather than on children. The sedentary behaviour research identified in this review was much more limited than that related to physical activity and covered only the first three of the five phases of the Behavioural Epidemiology Framework.

### Research Implications

The findings points towards the need for more and higher quality research. The following paragraphs describe the research required closely following the Behavioural Epidemiology Framework. Overall, the body of evidence included only a small number of prospective and cross-sectional studies which reported generally consistent associations between physical activity and sedentary behaviours and various health outcomes (Behavioural Epidemiology, Phase 1). Globally, there is extensive evidence on physical activity and life expectancy, cardiovascular disease, diabetes, cancer, mental health and bone health, but it largely originates from countries outside this study region [102, 103]. Examining the associations between patterns of physical activity and sedentary behaviours with various health outcomes in Arab populations should continue to address the knowledge gaps but future studies should employ rigorous methodologies including prospective study design and use objective measures of exposure to increase the quality of evidence available from this region (Behavioural Epidemiology, Phase 2).

Studies found in our review revealed overall low levels of participation in physical activity (particularly among young people), and high levels of sedentary behaviour (particularly among men and young people-Behavioural Epidemiology, Phase 3). Although the prevalence of physical activity among adolescent was generally higher than in adults, a large percentage of both adults and adolescents did not engage in sufficient amounts. Similar findings were observed in a global study of 34 countries [104]; however, the gender differences in the prevalence of physical activity, up to 40 percentage points more in boys than girls, is much larger in this review. Further studies using standardized methods of nationally representative samples are needed to monitor trends as well as to identify population variations and vulnerable groups. This point is particularly important since countries are expected to report on physical activity levels for both adult and adolescent populations as part of the WHO Global Plan of Action [105]. In addition, research on domain-specific physical activity for different populations groups is urgently needed to guide the development of targeted regionally appropriate interventions in light of regional trends like motorization and shifts in occupational patterns.

Studies exploring factors associated with physical activity (Behavioural Epidemiology, Phase 4) reported consistent associations with known correlates. Gender and age were consistently association with physical activity. Men were found to be more active than women and younger people more active than older people which is consistent with other countries [106, 107]. Only one study found that it was the older people more physical active [70]. Four studies in this review reported an inverse association of physical activity with education [70, 76, 90, 91], however, when findings from multiple countries are considered, the inconsistent evidence points to this correlate likely being more context and/or culture-specific [70]. A majority of the studies reporting on the individual-level correlates of physical activity were not guided by a formal conceptual model that could inform subsequent public-health approaches to behavioural change. While this might be construed as a limitation of the evidence that is available, addressing the need for more-basic descriptive data in the region should be a higher priority.

Few studies assessed the physical and policy environments across the four domains of active living (household, occupational, transport and recreation) [108]. Evidence on potential multiple levels of influence on physical activity (intrapersonal, perceived, social cultural, information, natural and policy environments) [108] exists for several Western countries; however, evidence for countries in this region is needed to understand the particular influences that may operate in Arab populations and their social and environmental contexts so as to inform policy and practice.

Only six studies reported the testing of population-based physical activity interventions [96–101] (Behavioural Epidemiology, Phase 5). Three studies reported positive results across different settings [96–98]. Given the importance of increasing physical activity and reducing sedentary behaviours as part of a comprehensive population based approach to the prevention of non-communicable disease [105], greater priority should be given to encouraging an increase in multidisciplinary intervention research to guide national policy and programs.

### Measurement of physical activity and sedentary behaviour

Underpinning the development of a strong body of research evidence on physical activity and sedentary behaviours is robust measurement of the exposure variables (Behavioral Epidemiology, Phase 2). This review revealed that many of the studies to date have employed a narrow understanding of physical activity behaviour, with their focus on “exercise” where this is a formal and structured activity. This is in contrast to the broader field of physical activity and public health which has adopted a wider view and includes all types of physical movement (such as walking, recreation, play, cycling to work, etc.) consistent with the WHO Global Recommendations [109].

Of particular concern this review revealed is the wide variability and quality in the measurement instruments used and the presentation of outcomes variables of exposure which severely limits within-and between-country comparisons. Except for those studies reporting use of two well established international measures (IPAQ and GPAQ), there was limited adoption of other valid and reliable tools to assess physical activity and sedentary behaviours, measures of the physical environment, self-reported cognitive, psychosocial measures and domain-specific measures [110]. There was one example of a formal multi-country initiative to develop, validate and utilize a specific physical activity measure for Arab populations [33]. It was the first collaborative project that assessed lifestyle-related variables in a large sample of adolescents from the nine countries of the Arab world. Most studies depended on self-report instruments with only a limited number employing objective instruments to assess either behaviours. Instruments such as accelerometers, pedometers, mobile phones or other electronic devices are increasingly being used across the international literature to address the limitations of self-report measures and are strongly recommended, especially in studies with children [111–113]. Accurate measurement is critical for policy development and necessary for population monitoring of trends over time and differences between populations. It is also essential for research aimed at programme evaluation of individual-based and population-based actions [110].

### Policy-relevant research

Although there were no studies that fit into the policy-related phase of the Behavioural Epidemiology Framework, we propose policy-relevant research though a critical review of our findings through the lens of international guidelines. To guide international efforts, recommendations on effective and feasible interventions have been provided on physical activity by the WHO in the Global Action Plan 2013–2020 [105]. Consistent with the 2011 report is the “Seven Investments to Promote Physical Activity” produced by Global Advocacy for Physical Activity (GAPA) [114–116]; intervention strategies are identified across seven key settings (school, transport, urban design, health services, mass media, sports and the community). Sustained public education campaigns using mass media to promote physical activity is a “best investment” recommended by both WHO [105] as well as GAPA [116, 117]. Based on the findings of this review, social marketing intervention efforts in the Arab Peninsular should be gender specific and target youth, especially girls and young women [17]. Campaigns should address the identified common barriers (i.e., limited time, lack of social support) [17, 18, 71–75, 86, 90, 92, 94, 95, 118–123] and cultural norms that place a low value on physical activity [17, 72, 119] and restrict women’s and girls’ participation in physical activity (i.e., cultural norm that limits women from walking/running on their own, or limited space within which to walk within the home compound) [17, 90, 93, 120]. Conducting the necessary formative and pilot research would help identify the promising strategies to then test in larger scale rigorous trials, including those integrating new media and community-based approaches [124].

A quarter of the population in these six countries are under 25 years of age [125, 126]. In addition, the prevalence of physical activity in adolescent populations is low, especially among girls, [57, 58, 65, 77–81]. Thus, interventions in the schools setting and implementation of a “whole of school” programme is a high priority for this region. The Health Promoting Schools (HPS) and Alharaka Baraka programmes [114, 117] provide a basis for developing and testing interventions as well as accelerating school-based action.

Limited capacity within health services to promote physical activity was identified as a key barrier [119, 127, 128]. Two interventions highlighted in this review provide some guidance on possible health services initiatives [98, 101]. Conducting research on how best to integrate promoting physical activity in health services should address gaps already identified. Suggestions have included intensive behaviour change interventions, training of healthcare workers and expanding preventive health services [119, 127–130].

Internationally there is increasing focus on the role of the physical environment [131–133] and consistent with other research, poor access was an identified barrier in this review [18, 71, 92, 119, 129, 134]. Better understanding the distribution, use and opportunities for improving the provision of sports programs and facilities more accessible to the general population, particularly girls and women is a priority for future research in this region. This should include better understanding of the role of informal recreational and sporting opportunities and facilities (such as hiking, biking, group exercise/aerobics, dance and martial arts).

Initiating research to examine the impact of urban planning and transport policy and practice in countries in the Arabian Peninsula is of importance. Research from elsewhere has identified that patterns of land-use, population density as well as the provision of adequate infrastructure to support ‘active transport’ (that is walking cycling and public transport) and optimal green and nature spaces are associated with higher levels of physical activity [131, 133, 135]. Given the rapid urban growth in this region as well as increasing levels of urban sprawl and motorization [136–138], research is needed to inform planning policy that is tailored to the culture and regional specific contexts. For example, encouraging more active transport and recreation needs to be understood within the context of the hot arid climate of the Arabian Peninsula [17, 18, 90, 93, 119, 120, 122, 139]. Research should explore the influence of climate, including seasonal variability (a known factor in other parts of the world) [140–142] and dust storms (a common event throughout the year) [143] on physical activity in the region. Initiating these lines of research may benefit from international collaborations which can build local research skills and capacity and benefit from extensive experience and protocols developed elsewhere [144, 145].

### Sedentary behaviour: an emerging field of research

The study of sedentary behaviour, relatively new globally, is only now beginning to receive the attention of researchers in the countries of the Arabian Peninsula. Only a third examined sedentary behaviour and the research was limited to phases 1, 2 and 3 of the Behavioural Epidemiology Framework. The proposed research agenda would be similar to that outlined globally; an ecological model of four domains of sedentary behaviour focusing specifically on domestic screen time, extended sitting time in workplaces and schools, and time spent sitting in cars -- not only to better understand their determinants but also in designing appropriate interventions [26].

### Strengths and Limitations

This is the first systematic review of physical activity and sedentary behaviour for this region and complements an earlier review of the prevalence of physical activity [146]. Adherence to the PRISMA statement and the use of three different search engines identified a substantial number of relevant studies. However, there were some limitations. First, our review was restricted to published studies in the English language. It is likely that additional studies such as government reports and scientific papers published in Arabic journals do exist but were not included. Second, the search was limited to only three multidisciplinary literature databases it is possible that additional databases may have identified more studies. Third, the variation in tools and methodologies as well as methodological quality limited within and cross-country comparability. These limitations may bias our view of the gaps in evidence and potential solutions. Nevertheless, the evidence that we have identified in this review is informative. Many evidence gaps remain in understanding how most appropriately to address physical activity and sedentary behaviour in the context of the increasing rates of NCDs in this unique region of the world.

## Conclusions

The epidemiological transition, including increasing life expectancy and changing mortality patterns, in the oil-producing countries of the Arabian Peninsula has taken only 50 years; a timeframe much more rapid than for many other high-income countries. The rapidly rising prevalence of NCDs and increased susceptibility of the population to these diseases have dire consequences to future generations. The predicted trends and future burden on health care systems demands that public health action be more interventionist than those in developed countries (James PT: WHO Mission Report: Nutrition Planning for Health in 2050, unpublished). Given the low levels of physical activity in the Arabian Peninsula and high levels of sedentary behaviour, a much stronger evidence base is needed to guide action than is currently available.

Policy relevant research should be undertaken by interdisciplinary teams of policy makers and researchers [26, 108]. Guided by the Behavioural Epidemiology Framework, priority research includes examining these behaviours across the four domains (household, work, transport and leisure). Following the ecological model and using standard assessment tools will improve the quality of research. For the short-term, the most feasible and priority intervention research is in the education, health and sports sectors, especially targeting women and young people. Adapting and testing international models and assessing some of the positive examples in the Arabian Peninsula [114, 115], can help guide and/or refine current policy and practice [147].

## Additional file

Additional file 1: Quality Assessment of Studies on Physical Activity and Sedentary Behaviour in the Arabian Peninsula. (DOCX 124 kb)
