# Supplementary material for: A systematic review of physical activity and sedentary behaviour research in the oil-producing countries of the Arabian Peninsula
Source: BMC Public Health. 2016 Sep 21;16:1003. doi: 10.1186/s12889-016-3642-4 (PMC5031342; doi:10.1186/s12889-016-3642-4)
Supplement: Additional file 1: — Quality Assessment of Studies on Physical Activity and Sedentary Behaviour in the Arabian Peninsula. (DOCX 124 kb) [file 12889_2016_3642_MOESM1_ESM.docx]

**Supplementary Table. Quality Assessment of Studies on Physical Activity and Sedentary Behaviour in the Arabian Peninsula**

| **No.** | **Authors (Year)** | **Published Table(s)** | **Quality Assessment^a^** | | | | | | **Tools^b^** | |
| --- | --- | --- | --- | --- | --- | --- | --- | --- | --- | --- |
|  |  |  | **Eligibility** | **Randomization**  **(Q: Sample selection)** | **Sample Description** | **PA/SB** | **Co-variates**  **(Q: Analysis and Interpretation** | **Total** | **Physical Activity (Type)c** | **Sedentary Behavior (Type)** |
| 1 | Abd-El-Fattah SM (2015)[1] | - | 1 | 0 | 1 | 1 | 0 | 3 | PASESA |  |
| 2 | Abdi S, Sadiya A, et al (2015)[2] | 2 | 1 | 1 | 1 | 1 | 0 | 4 | PA intervention |  |
| 3 | Abduelkarem A and Sackville M (2009)[3] | 5 | 0 | 0 | 1 | 0 | 0 | 1 | SDSCA (Total) |  |
| 4 | AboZaid HA and Farahat FM (2010)[4] | 4 | 0 | 0 | 1 | 1 | 0 | 2 | IPAQ-short (Total) |  |
| 5 | Al Daghri NM, Alokail MS, et al (2015)[5] | 2 | 1 | 1 | 1 | 1 | 1 | 5 | FIT index (Total) |  |
| 6 | Al Junaibi A, Abdulle A, et al (2012)[6] | 2 | 1 | 1 | 1 | 1 | 0 | 4 | IPAQ-short (Walking, Total) | IPAQ-short (TV/Computer, Total sitting) |
| 7 | Al Saif A and Alsenany S (2015)[7] | 2 | 0 | 0 | 0 | 1 | 0 | 1 | Exercise intervention |  |
| 8 | Al Saweer A, Salehi S, et al (2015)[8] | 2 | 0 | 1 | 0 | 1 | 0 | 2 | Exercise intervention |  |
| 9 | Al Thani MH, Al Thani A, et al (2015) [9] | 3 | 1 | 1 | 1 | 1 | 1 | 5 | GPAQ (Total) | GPAQ (Total) |
| 10 | Al-Doghether M, Al-Tuwijri A, et al (2007)[10] | 4 | 0 | 1 | 1 | 0 | 0 | 2 | Local tool (ND) |  |
| 11 | Al-Eisa ES and Al-Sobayel HI (2012)[11] | 4 | 1 | 0 | 1 | 1 | 0 | 3 | Pedometer (Total) |  |
| 12 | Al-Eisa E, Alghadir AH, et al (2016) [12] | 2 | 1 | 1 | 1 | 1 | 0 | 4 | Exercise intervention |  |
| 13 | Al-Eisa E, Buragadda S, et al (2014) [13] | 2 | 1 | 1 | 1 | 1 | 0 | 4 | Pedometer |  |
| 14 | Al-Eisa E, Al-Rushud A, et al (2016)[14] | 5 | 1 | 1 | 1 | 1 | 0 | 4 | Exercise intervention |  |
| 15 | Alghadir AH, Gabr SA, et al (2016) [15] | 2 | 0 | 1 | 1 | 1 | 0 | 3 | Exercise intervention |  |
| 16 | Alghadir AH, Gabr SA, et al (2015) [16] | 2 | 0 | 1 | 1 | 1 | 0 | 3 | Exercise intervention |  |
| 17 | Al-Ghawi A and Uaug R (2009)[17] | 4 | 0 | 1 | 1 | 0 | 0 | 2 | Local tool (Exercise, Attitude to counsel on PA) |  |
| 18 | Al-Ghimlas F, Subbramaniam K, et al (2014)[18] | 2 | 1 | 1 | 1 | 1 | 0 | 4 | Exercise intervention |  |
| 19 | Al-Haifi AR, Al-Fayez MA, et al (2013)[19] | 2 | 1 | 1 | 1 | 1 | 0 | 4 | ATLS (Total) | ATLS (TV/computer) |
| 20 | Al-Haifi AA, AlMajed HT, et al (2016)[20] | 2 | 1 | 1 | 1 | 1 | 1 | 5 | ATLS (Total) | ATLS (TV/computer) |
| 21 | Al-Hamdan NA, Al-Zalabani, et al (2012)[21] | 2 | 0 | 1 | 1 | 1 | 0 | 3 | GPAQ (Total) |  |
| 22 | Al-Hazzaa HM and Al-Rasheedi AA (2007)[22] | 3 | 0 | 1 | 1 | 1 | 1 | 4 | Pedometer | Local (TV) |
| 23 | Al-Hazzaa HM, Abahussain NA, et al (2011)[23] | 3 | 1 | 1 | 1 | 1 | 1 | 5 | ATLS (Total) | ATLS (TV/computer) |
| 24 | Al-Hazzaa HM, Abahussain NA, et al (2012)[24] | 2 | 1 | 1 | 1 | 1 | 1 | 5 | ATLS (Total) | ATLS (TV/computer) |
| 25 | Al-Hazzaa HM, Alahmadi, et al (2015)[25] | 3, 4 | 1 | 1 | 1 | 1 | 1 | 5 | ATLS (Total) | ATLS (TV/computer) |
| 26 | Al-Hazzaa HM, Al-Nakeeb Y, et al (2013)[26] | 3, 4 | 1 | 1 | 1 | 1 | 1 | 5 | ATLS (Total) | ATLS (TV/computer) |
| 27 | Al-Hazzaa HM, Al-Sobayet HI, al (2011)[27] | - | 0 | 1 | 1 | 1 | 1 | 4 | ATLS (Total), Pedometer | ATLS (TV/computer) |
| 28 | Al-Hazzaa HM, Al-Sobayel HI, et al (2013)[28] | 3 | 1 | 1 | 1 | 1 | 1 | 5 | ATLS (Total) | ATLS (TV/computer) |
| 29 | Al-Hazzaa HM, Musaiger AO, et al (2013)[29] | 2 | 1 | 1 | 1 | 1 | 1 | 5 | ATLS (Total) | ATLS (TV/computer) |
| 30 | Al-Hazzaa HM (2007)[30] | 3, 4 | 0 | 1 | 1 | 1 | 1 | 4 | IPAQ-short (Total) |  |
| 31 | Ali HI, Bernsen RM, et al (2008 -Q)[31] | 4 | 1 | 1 | 1 | 0 | 1 | 4 |  |  |
| 32 | Ali HI, Baynouna LM, et at (2010 - Q)[32] | 4 | 1 | 1 | 1 | 0 | 1 | 4 |  |  |
| 33 | Alkahtani MG, Elkilany AM, et al (2015)[33] | 4 | 1 | 0 | 1 | 1 | 1 | 4 | Accelerometer |  |
| 34 | Al-Kilani H, Waly M, et al (2012)[34] | 2 | 1 | 1 | 1 | 0 | 0 | 3 | Local Tool (ND) |  |
| 35 | Allafi A, Al-Haifi AR, et al. (2013)[35] | 3 | 1 | 1 | 1 | 1 | 0 | 4 | ATLS (Total) | ATLS (TV/computer) |
| 36 | Allam AR, Taha IM, et al (2012)[36] | 3 | 1 | 1 | 1 | 1 | 0 | 4 | IPAQ (ND) |  |
| 37 | Al-Mahroos F and Al-Roomi K (2001)[37] | 2 | 0 | 1 | 1 | 0 | 1 | 3 | WHO Heart and Health (Work, walking/cycling) | WHO Heart and Health (TV) |
| 38 | Al-Majwal, AM (2015)[38] | 2, 4 | 0 | 1 | 1 | 1 | 1 | 4 | Local tool (Total) |  |
| 39 | Al-Nakeeb Y, Lyons M, et al (2012)[39] | 2, 3 | 0 | 1 | 1 | 1 | 0 | 3 | ATLS (Walking, Total) | ATLS (TV/computer) |
| 40 | Al-Nozha MM, Al-Hazzaa HM, et al (2007)[40] | 2, 3, 4 | 0 | 1 | 1 | 1 | 0 | 3 | Local tool (Total) |  |
| 41 | Al-Nuaim AA, Al-Nakeeb Y, et al (2012)[41] | 2, 3 | 0 | 0 | 1 | 1 | 0 | 2 | ATLS (Total) | ATLS (TV/computer) |
| 42 | Al-Otaibi HH (2013)[42] | 4 | 1 | 0 | 1 | 0 | 0 | 2 | Local tool (Total, Stages of change, barriers and self-efficacy) |  |
| 43 | Alqahtani N and Scott J (2015)[43] | 2 | 0 | 1 | 0 | 1 | 1 | 3 | Local tool (Total) | Local tool (screen time) |
| 44 | AlQuaiz AM and Tayel SA (2009)[44] | 4 | 1 | 1 | 1 | 1 | 0 | 4 | Adapted CDC (Total, Barriers) |  |
| 45 | Al-Rafaee SA and Al-Hazzaa HM (2001)[45] | 4 | 0 | 1 | 1 | 0 | 0 | 2 | Local tool (ND) |  |
| 46 | Alrashidi M, Shahwan-Akl L, et al (2015)[46] | 2 | 1 | 1 | 1 | 1 | 0 | 4 |  | Local tool (TV/video) |
| 47 | Al-Sobayel H, Al-Hazzaa HM, et al (2015)[47] | 4 | 1 | 1 | 1 | 1 | 1 | 5 | ATLS (Total) |  |
| 48 | Alsubaie AS and Omer EO (2015)[48] | 4 | 1 | 1 | 1 | 0 | 0 | 3 | 30-day recall (ND) |  |
| 49 | Al-Thani, MH, Al-Thani AA, et al (2015)[49] | 2 | 1 | 1 | 1 | 1 | 1 | 5 | GPAQ (Total) |  |
| 50 | Amin TT, Suleman W, et al (2011)[50] | 4 | 1 | 1 | 1 | 1 | 1 | 5 | GPAQ (Total) |  |
| 51 | Amin TT, Al Khoudair AS, et al (2012)[51] | 4 | 1 | 1 | 1 | 1 | 1 | 5 | GPAQ (Total) |  |
| 52 | Ardawi MM, Rouzi AA, et al (2012)[52] | 2 | 1 | 1 | 1 | 1 | 0 | 4 | Local tool (ND), Accelerometer and exercise intervention |  |
| 53 | Awadalla NJ, Aboelyazed AE, et al (2014)[53] | 3, 4 | 1 | 1 | 1 | 1 | 0 | 4 | IPAQ short (Total)  Local tool (barriers) |  |
| 54 | Banday AH, Want FA, et al (2015)[54] | 3 | 1 | 1 | 1 | 1 | 0 | 4 | GPAQ (Total) |  |
| 55 | Barss P, Grivna M, et al (2008)[55] | 5 | 1 | 0 | 1 | 0 | 0 | 2 | Local tool (ND) |  |
| 56 | Basulaiman M, El Bcheraoui C, et al (2014)[56] | 2 | 1 | 1 | 1 | 1 | 1 | 5 | IPAQ (Total) | IPAQ (Total) |
| 57 | Berger G and Peerson A (2009 - Q)[57] | 4 | 1 | 0 | 1 | 0 | 1 | 3 |  |  |
| 58 | Carter AO, Elzubeir M, et al (2003)[58] | 3 | 1 | 0 | 1 | 0 | 0 | 2 | Nurses Healthy Study II (ND) |  |
| 59 | Daradkeh G, Al Muhannadi A, et al (2015)[59] | 4 | 1 | 1 | 1 | 0 | 0 | 3 | Local tool (Total) |  |
| 60 | Donnelly TT, Al Suwaidi J, et al (2012 - Q)[60] | 4 | 1 | 1 | 1 | 1 | 1 | 5 |  |  |
| 61 | Duncan MJ, Al-Hazzaa HM, et al (2015)[61] | 4 | 1 | 1 | 1 | 1 | 1 | 5 | ATLS (Total) | ATLS (TV/computer) |
| 62 | El Bcheraoui C, Memish ZA, et al (2014)[62] | 2 | 1 | 1 | 1 | 1 | 1 | 5 | IPAQ (Total) | IPAQ (TV) |
| 63 | El-Aty M, Mabry R, et al (2014)[63] | 2, 3 | 1 | 1 | 1 | 1 | 1 | 5 | GPAQ (Total) | GPAQ (Total) |
| 64 | El-Ghazali S, Ibrahim JM, et al (2010)[64] | 2 | 0 | 1 | 1 | 0 | 0 | 2 | Local tool (Total) | Local Tool (TV/computer) |
| 65 | Farghaly NF, Ghazali BM, et al (2007)[65] | 3 | 0 | 1 | 1 | 0 | 0 | 2 | Local Tool (Total) | Local Tool (TV) |
| 66 | Faris MA, Epuru S, et al (2015)[66] | 4 | 1 | 1 | 1 | 0 | 1 | 4 | Local tool (Total) |  |
| 67 | Gawwad ES (2008)[67] | 4 | 0 | 1 | 1 | 0 | 0 | 2 | Local Tool (Total, Stages of Change, Benefits and Barriers, Self-Efficacy) | Local Tool |
| 68 | Gharib NM and Rasheed P (2008) [68] | 3 | 0 | 1 | 1 | 0 | 1 | 3 | Local Tool (Total) | Local Tool (TV/computer) |
| 69 | Grant N, Gibbs T, et al (2007)[69] | 5 | 1 | 0 | 1 | 0 | 0 | 2 | Local Tool (ND) |  |
| 70 | Hashim R and Al-Ali K (2013)[70] | 4 | 1 | 1 | 1 | 0 | 0 | 3 | Local Tool (ND) |  |
| 71 | Hegazy AM, Salama BM, et al (2015)[71] | 2 | 1 | 1 | 1 | 0 | 1 | 4 | Local tool (ND) |  |
| 72 | Kerkadi A, Abo Elnaga N, et al (2005)[72] | 3 | 0 | 1 | 1 | 0 | 1 | 3 | Local Tool (Total) | Local Tool (TV) |
| 73 | Khalaf A, Ekblom O, et al (2013)[73] | 3 | 0 | 1 | 1 | 1 | 1 | 4 | ATLS (Total) | ATLS (TV/computer) |
| 74 | Kilani H Al-Hazzaa H, et al (2013)[74] | 2 | 1 | 1 | 1 | 1 | 0 | 4 | ATLS (Total) | ATLS (TV/computer) |
| 75 | Kneffel Z, Goebel, R, et al (2015)[75] | 2 | 1 | 1 | 1 | 1 | 0 | 4 | PA intervention |  |
| 76 | Koura MR, Al-Dabal BK, et al (2012)[76] | 3 | 0 | 1 | 1 | 0 | 0 | 2 | GPAQ (Total) |  |
| 77 | Mabry RM, Winkler EA, et al (2012)[77] | 2 | 1 | 1 | 1 | 1 | 1 | 5 | GPAQ (Work, Transport, Leisure) | GPAQ (Total) |
| 78 | Mabry RM, Winkler EA, et al (2012)[78] | 3, 4 | 1 | 1 | 1 | 1 | 1 | 5 | GPAQ (Work, Transport, Leisure, Total) | GPAQ (Total) |
| 79 | Mabry RM, Al-Busaidi ZQ, et al (2013 - Q)[79] | 4 | 1 | 1 | 1 | 1 | 1 | 5 |  |  |
| 80 | Mahfouz AA, Abdelmoneim I, et al (2008)[80] | 3 | 0 | 1 | 1 | 0 | 1 | 3 | Adapted CDC Adolescent Health Survey (Total) | Adapted CDC Adolescent Health Survey (TV) |
| 81 | Mahfouz AA, Shatoor AS, et al (2011)[81] | 3 | 0 | 1 | 1 | 0 | 1 | 3 | Adapted CDC Adolescent Health Survey (Total) | Adapted CDC Adolescent Health Survey (TV) |
| 82 | Memish, ZA, El Bcheraoui C, et al (2014)[82] | 2 | 1 | 1 | 1 | 1 | 1 | 5 | IPAQ (Total) |  |
| 83 | Midhet FM, Sharaf FK. (2011)[83] | 5 | 0 | 1 | 1 | 0 | 1 | 3 | Local Tool (Exercise) |  |
| 84 | Moradi-Lakeh M, El Bcheraoui C, et al (2013)[84] | 2 | 1 | 1 | 1 | 1 | 1 | 5 | IPAQ (Total) | IPAQ (TV/computer) |
| 85 | Muhairi SJ, Mehairi A, et al (2013)[85] | 2 | 1 | 1 | 1 | 0 | 1 | 4 | IPAQ-short (Total) | IPAQ-short (TV/computer) |
| 56 | Musaiger AO, Al-Kandari FI, et al (2014)[86] | 4 | 0 | 0 | 1 | 0 | 0 | 1 | Local tool (Barriers) |  |
| 87 | Musaiger AO and Zagzoog N (2013)[87] | 3 | 0 | 1 | 1 | 0 | 0 | 2 | Local tool (Exercise) | Local (TV/Internet) |
| 88 | Platat C and Jarrar A (2011)[88] | - | 0 | 0 | 1 | 1 | 0 | 2 | QAPACE, pedometer |  |
| 89 | Rouzi AA, Al-Sibani SA, et al (2011)[89] | 2 | 1 | 1 | 1 | 0 | 1 | 4 | 7-day Physical Activity Recall Scale (Total) |  |
| 90 | Sadiya A, Abdi S, et al (2016)[90] | 2 | 1 | 1 | 1 | 1 | 1 | 5 | Pedometer  Lifestyle intervention including exercise |  |
| 91 | Salman RA and Al-Rubeaan KA (2009)[91] | 4 | 1 | 0 | 1 | 0 | 1 | 3 | Local tool (Total) |  |
| 92 | Serour M, Alqhenaei H, et al (2007)[92] | 4 | 1 | 0 | 1 | 1 | 0 | 3 | IPAQ-short (Total) |  |
| 93 | Shah SM, Loney T, et al (2015)[93] | 2 | 1 | 1 | 1 | 0 | 1 | 4 | GPAQ (Total) |  |
| 94 | Sharaf F (2010)[94] | 5 | 1 | 0 | 0 | 0 | 1 | 2 | Local tool (Exercise) |  |
| 95 | Sulaiman N, Hamdan A, et al (2009 - Q)[95] | 4 | 1 | 1 | 1 | 0 | 1 | 4 |  |  |
| 96 | Taha AZ (2008)[96] | 4 | 1 | 1 | 1 | 0 | 1 | 4 | Local tool (Exercise) |  |
| 97 | Tomar RH, Hashim MH, et al (2013)[97] | 2 | 1 | 1 | 1 | 1 | 0 | 4 | Exercise intervention |  |
| 98 | Tuffaha M, El Bcheraoui C, et al (2015)[98] | 2 | 1 | 1 | 1 | 1 | 1 | 5 | IPAQ (Total) |  |
| 99 | Yousef S, Eapen V, et al (2014)[99] | 2, 3 | 1 | 1 | 1 | 0 | 0 | 3 |  | Childhood Behavioral Checklist (TV) |
| 100 | Youssef RM, Al-Shafie K (2013)[100] | 3 | 1 | 1 | 1 | 1 | 1 | 5 | Local Tool (Total, Barriers | Local (TV/computer) |

1. Quality assessment: Except for qualitative studies, all studies were assigned “1” for each of the following criteria if they provided an adequate description: 1. Eligibility, 2. Randomization of participant selection or assignment for case-control studies, iii. Study sample (including number/size, age and gender), 3. Measurement of physical activity includes duration, intensity and frequency (alternatively, for studies in Table 1 and 4 studies should include an adequate description of physical activity in-line with objectives of the study; for studies in Table 2, they should also use the WHO recommendation on physical activity….) and/or measurement of sedentary behavior, 5. Co-variates included in the data analysis. Qualitative studies (Q) were assigned “1” for each of the following criteria if they provided an adequate description: 1. Eligibility, 2. Sample Selection, 3. Study sample (including number/size, age and gender, 4. Recognition of standard definition for physical activity and/or sedentary behavior, 5. Analysis/interpretation
2. Tools: Published tool (GPAQ: Global Physical Activity Questionnaire; IPAQ: International Physical Activity Questionnaire; ATLS: Arab Teens Lifestyle Student Questionnaire, PASESA: Physical Activity Self-Efficacy Scale for Adolescents, SDSCA: Summary of Diabetes Self-Care Activities) or locally developed tool
3. ND: Tools and/or type of PA or SB not adequately described

References:

1. *Abd-El-Fattah SM: **Rasch Rating Scale Analysis of the Arabic Version of the Physical Activity Self-Efficacy Scale for Adolescents: A Social Cognitive Perspective**. *Psychology* 2015, **06**(16):2161-2180.

2. *Abdi S, Sadiya A, Ali S, Varghese S, Abusnana S: **Behavioural Lifestyle Intervention Study (BLIS) in patients with type 2 diabetes in the United Arab Emirates: A randomized controlled trial**. *BMC Nutrition* 2015, **1**(1):1.

3. *Abduelkarem A, Sackville M: **Changes of some Health Indicators in Patients with Type 2 Diabetes: A Prospective Study in three Community Pharmacies in Sharjah, United Arab Emirates**. *Libyan J Med* 2009, **4**(1):31-36.

4. *AboZaid HA, Farahat FM: **Physical activity profile among patients attending family medicine clinics in western Saudi Arabia**. *Saudi Med J* 2010, **31**(4):428-433.

5. *Al-Daghri NM, Alokail MS, Rahman S, Amer OE, Al-Attas OS, Alfawaz H, Tripathi G, Sabico S, Chrousos GP, McTernan PG *et al*: **Habitual physical activity is associated with circulating irisin in healthy controls but not in subjects with diabetes mellitus type 2**. *Eur J Clin Invest* 2015, **45**(8):775-781.

6. *Al Junaibi A, Abdulle A, Sabri S, Hag-Ali M, Nagelkerke N: **The prevalence and potential determinants of obesity among school children and adolescents in Abu Dhabi, United Arab Emirates**. *Int J Obes (Lond)* 2012, **37**(1):68-74.

7. *Al Saif A, Alsenany S: **Aerobic and anaerobic exercise training in obese adults**. *J Phys Ther Sci* 2015, **27**(6):1697-1700.

8. *Al Saweer A, Salehi S, Al Tiho M, Al Saffar B, Al Amer M: **Interventional Program for Teenagers' Obesity**. *Bahrain Medical Bulletin* 2015, **37**(2):109-+.

9. *Al Thani M, Al Thani A, Al-Chetachi W, Al Malki B, Khalifa SAH, Bakri AH, Hwalla N, Nasreddine L, Naja F: **Lifestyle Patterns Are Associated with Elevated Blood Pressure among Qatari Women of Reproductive Age: A Cross-Sectional National Study**. *Nutrients* 2015, **7**(9):7593-7615.

10. *Al-Doghether M, Al-Tuwijri A, Khan A: **Obstacles to preventive intervention. Do physicians' health habits and mind-set towards preventive care play any role?** *Saudi Med J* 2007, **28**(8):1269-1274.

11. *Al-Eisa ES, Al-Sobayel HI: **Physical Activity and Health Beliefs among Saudi Women**. *J Nutr Metab* 2012, **2012**:642187.

12. *Al-Eisa E, Alghadir AH, Gabr SA, Iqbal ZA: **Exercise intervention as a protective modulator against metabolic disorders in cigarette smokers**. *J Phys Ther Sci* 2016, **28**(3):983-991.

13. *Al-Eisa E, Buragadda S, Melam GR: **Association between physical activity and psychological status among Saudi female students**. *BMC Psychiatry* 2014, **14**:238.

14. *Al-Eisa E, Al-Rushud A, Alghadir A, Anwer S, Al-Harbi B, Al-Sughaier N, Al-Yoseef N, Al-Otaibi R, Al-Muhaysin HA: **Effect of Motivation by "Instagram" on Adherence to Physical Activity among Female College Students**. *Biomed Res Int* 2016, **2016**:1546013.

15. *Alghadir AH, Gabr SA, Al-Eisa ES, Alghadir MH: **Correlation between bone mineral density and serum trace elements in response to supervised aerobic training in older adults**. *Clin Interv Aging* 2016, **11**:265-273.

16. *Alghadir AH, Gabr SA, Aly FA: **The effects of four weeks aerobic training on saliva cortisol and testosterone in young healthy persons**. *J Phys Ther Sci* 2015, **27**(7):2029-2033.

17. *Al-Ghawi A, Uauy R: **Study of the knowledge, attitudes and practices of physicians towards obesity management in primary health care in Bahrain**. *Public Health Nutr* 2009, **12**(10):1791-1798.

18. *Al-Ghimlas F, Subbramaniam K, Al-Owaish O, Bilas MT, Behbehani K: **The Effects of Supervised Exercise Program on Health-Related Physical Fitness in Kuwait**. *British Journal of Medicine and Medical Research* 2014, **4**(32):5083.

19. *Al-Haifi AR, Al-Fayez MA, Al-Athari BI, Al-Ajmi FA, Allafi AR, Al-Hazzaa HM, Musaiger AO: **Relative contribution of physical activity, sedentary behaviors, and dietary habits to the prevalence of obesity among Kuwaiti adolescents**. *Food Nutr Bull* 2013, **34**(1):6-13.

20. *Al-Haifi AA, AlMajed HT, Al-Hazzaa HM, Musaiger AO, Arab MA, Hasan RA: **Relative Contribution of Obesity, Sedentary Behaviors and Dietary Habits to Sleep Duration Among Kuwaiti Adolescents**. *Glob J Health Sci* 2016, **8**(1):107-117.

21. *Al-Hamdan NA, Al-Zalabani AH, Saeed AA: **Comparative study of physical activity of hypertensives and normotensives: A cross-sectional study of adults in Saudi Arabia**. *J Family Community Med* 2012, **19**(3):162-166.

22. *Al-Hazzaa HM, Al-Rasheedi AA: **Adiposity and physical activity levels among preschool children in Jeddah, Saudi Arabia**. *Saudi Med J* 2007, **28**(5):766-773.

23. *Al-Hazzaa HM, Abahussain NA, Al-Sobayel HI, Qahwaji DM, Musaiger AO: **Physical activity, sedentary behaviors and dietary habits among Saudi adolescents relative to age, gender and region**. *Int J Behav Nutr Phys Act* 2011, **8**:140.

24. *Al-Hazzaa HM, Abahussain NA, Al-Sobayel HI, Qahwaji DM, Musaiger AO: **Lifestyle factors associated with overweight and obesity among Saudi adolescents**. *BMC Public Health* 2012, **12**:354.

25. *Al-Hazzaa HM, Alahmadi MA, Al-Sobayel HI, Abahussain NA, Qahwaji DM, Musaiger AO: **Patterns and determinants of physical activity among saudi adolescents**. *J Phys Act Health* 2014, **11**(6):1202-1211.

26. *Al-Hazzaa HM, Al-Nakeeb Y, Duncan MJ, Al-Sobayel HI, Abahussain NA, Musaiger AO, Lyons M, Collins P, Nevill A: **A cross-cultural comparison of health behaviors between Saudi and British adolescents living in urban areas: gender by country analyses**. *Int J Environ Res Public Health* 2013, **10**(12):6701-6720.

27. *Al-Hazzaa HM, Al-Sobayel HI, Musaiger AO: **Convergent validity of the Arab Teens Lifestyle Study (ATLS) physical activity questionnaire**. *Int J Environ Res Public Health* 2011, **8**(9):3810-3820.

28. *Al-Hazzaa HM, Al-Sobayel HI, Abahussain NA, Qahwaji DM, Alahmadi MA, Musaiger AO: **Association of dietary habits with levels of physical activity and screen time among adolescents living in Saudi Arabia**. *J Hum Nutr Diet* 2013.

29. *Al-Hazzaa HM, Musaiger AO, Abahussain NA, Al-Sobayel HI, Qahwaji DM: **Lifestyle correlates of self-reported sleep duration among Saudi adolescents: a multicentre school-based cross-sectional study**. *Child Care Health Dev* 2013.

30. *Al-Hazzaa HM: **Health-enhancing physical activity among Saudi adults using the International Physical Activity Questionnaire (IPAQ)**. *Public Health Nutr* 2007, **10**(1):59-64.

31. *Ali HI, Bernsen RM, Baynouna LM: **Barriers to weight management among Emirati women: a qualitative investigation of health professionals' perspectives**. *Int Q Community Health Educ* 2008, **29**(2):143-159.

32. *Ali HI, Baynouna LM, Bernsen RM: **Barriers and facilitators of weight management: perspectives of Arab women at risk for type 2 diabetes**. *Health Soc Care Community* 2010, **18**(2):219-228.

33. *Alkahtani SA, Elkilany AM, Al-Mohannadi AS, AlDuhIshy AM: **Relationship Between Self-Reported Dietary Intake and Measured Physical Activity among Male Students in the Preparatory Year in University of Dammam in Saudi Arabia**. *Current Research in Nutrition and Food Science Journal* 2015, **3**(2):130-139.

34. *Al-Kilani H, Waly M, Yousef R: **Trends of Obesity and Overweight among College Students in Oman: A cross sectional study**. *Sultan Qaboos Univ Med J* 2012, **12**(1):69-76.

35. *Allafi A, Al-Haifi AR, Al-Fayez MA, Al-Athari BI, Al-Ajmi FA, Al-Hazzaa HM, Musaiger AO, Ahmed F: **Physical activity, sedentary behaviours and dietary habits among Kuwaiti adolescents: gender differences**. *Public Health Nutr* 2013:1-8.

36. *Allam AR, Taha IM, Al-Nozha OM, Sultan IE: **Nutritional and health status of medical students at a university in Northwestern Saudi Arabia**. *Saudi Med J* 2012, **33**(12):1296-1303.

37. *Al-Mahroos F, Al-Roomi K: **Obesity among adult Bahraini population: impact of physical activity and educational level**. *Ann Saudi Med* 2001, **21**(3-4):183-187.

38. *Almajwal AM: **Correlations of Physical Activity, Body Mass Index, Shift Duty, and Selected Eating Habits among Nurses in Riyadh, Saudi Arabia**. *Ecol Food Nutr* 2015, **54**(4):397-417.

39. *Al-Nakeeb Y, Lyons M, Collins P, Al-Nuaim A, Al-Hazzaa H, Duncan MJ, Nevill A: **Obesity, physical activity and sedentary behavior amongst British and Saudi youth: a cross-cultural study**. *Int J Environ Res Public Health* 2012, **9**(4):1490-1506.

40. *Al-Nozha MM, Al-Hazzaa HM, Arafah MR, Al-Khadra A, Al-Mazrou YY, Al-Maatouq MA, Khan NB, Al-Marzouki K, Al-Harthi SS, Abdullah M *et al*: **Prevalence of physical activity and inactivity among Saudis aged 30-70 years. A population-based cross-sectional study**. *Saudi Med J* 2007, **28**(4):559-568.

41. *Al-Nuaim AA, Al-Nakeeb Y, Lyons M, Al-Hazzaa HM, Nevill A, Collins P, Duncan MJ: **The Prevalence of Physical Activity and Sedentary Behaviours Relative to Obesity among Adolescents from Al-Ahsa, Saudi Arabia: Rural versus Urban Variations**. *J Nutr Metab* 2012, **2012**:417589.

42. *Al-Otaibi HH: **Measuring Stages of Change, Perceived Barriers and Self efficacy for Physical Activity in Saudi Arabia**. *Asian Pac J Cancer Prev* 2013, **14**(2):1009-1016.

43. *Alqahtani N, Scott J, S U: **Physical Activity and Sedentary Behaviors as Risk Factors of Obesity among Rural Adolescents**. *Journal of Child and Adolescent Behaviour* 2015, **2015**.

44. *AlQuaiz AM, Tayel SA: **Barriers to a healthy lifestyle among patients attending primary care clinics at a university hospital in Riyadh**. *Ann Saudi Med* 2009, **29**(1):30-35.

45. *Al-Rafaee SA, Al-Hazzaa HM: **Physical activity profile of adult males in Riyadh City**. *Saudi Med J* 2001, **22**(9):784-789.

46. *Alrashidi M, Shahwan-Akl L, James J, Jones L: **Contributing factors to childhood overweight and obesity in Kuwait**. *International Journal of Health Sciences* 2015, **3**(1):133-155.

47. *Al-Sobayel H, Al-Hazzaa HM, Abahussain NA, Qahwaji DM, Musaiger AO: **Gender differences in leisure-time versus non-leisure-time physical activity among Saudi adolescents**. *Ann Agric Environ Med* 2015, **22**(2):344-348.

48. *Alsubaie AS, Omer EO: **Physical Activity Behavior Predictors, Reasons and Barriers among Male Adolescents in Riyadh, Saudi Arabia: Evidence for Obesogenic Environment**. *Int J Health Sci (Qassim)* 2015, **9**(4):400-408.

49. *Al-Thani MH, Al-Thani AA, Al-Chetachi WF, Khalifa SAH, Akram H, Poovelil BV, Almalki BA, Bakri AH, Arora P, Badawi A: **Dietary and Nutritional Factors Influencing Obesity in Qatari Adults and the Modifying Effect of Physical Activity**. *J Obes Weight-Loss Medic* 2015, **1**(1).

50. *Amin TT, Suleman W, Ali A, Gamal A, Al Wehedy A: **Pattern, prevalence, and perceived personal barriers toward physical activity among adult Saudis in Al-Hassa, KSA**. *J Phys Act Health* 2011, **8**(6):775-784.

51. *Amin TT, Al Khoudair AS, Al Harbi MA, Al Ali AR: **Leisure time physical activity in Saudi Arabia: prevalence, pattern and determining factors**. *Asian Pac J Cancer Prev* 2012, **13**(1):351-360.

52. *Ardawi MS, Rouzi AA, Qari MH: **Physical activity in relation to serum sclerostin, insulin-like growth factor-1, and bone turnover markers in healthy premenopausal women: a cross-sectional and a longitudinal study**. *J Clin Endocrinol Metab* 2012, **97**(10):3691-3699.

53. *Awadalla NJ, Aboelyazed AE, Hassanein MA, Khalil SN, Aftab R, Gaballa, II, Mahfouz AA: **Assessment of physical inactivity and perceived barriers to physical activity among health college students, south-western Saudi Arabia**. *East Mediterr Health J* 2014, **20**(10):596-604.

54. *Banday AH, Want FA, Alris FF, Alrayes MF, Alenzi MJ: **A Cross-sectional Study on the Prevalence of Physical Activity Among Primary Health Care Physicians in Aljouf Region of Saudi Arabia**. *Mater Sociomed* 2015, **27**(4):263-266.

55. *Barss P, Grivna M, Al-Maskari F, Kershaw G: **Strengthening public health medicine training for medical students: development and evaluation of a lifestyle curriculum**. *Med Teach* 2008, **30**(9-10):e196-218.

56. *Basulaiman M, El Bcheraoui C, Tuffaha M, Robinson M, Daoud F, Jaber S, Mikhitarian S, Wilson S, Memish ZA, Al Saeedi M *et al*: **Hypercholesterolemia and its associated risk factors-Kingdom of Saudi Arabia, 2013**. *Ann Epidemiol* 2014, **24**(11):801-808.

57. *Berger G, Peerson A: **Giving young Emirati women a voice: participatory action research on physical activity**. *Health Place* 2009, **15**(1):117-124.

58. *Carter AO, Elzubeir M, Abdulrazzaq YM, Revel AD, Townsend A: **Health and lifestyle needs assessment of medical students in the United Arab Emirates**. *Med Teach* 2003, **25**(5):492-496.

59. *Daradkeh G, Al Muhannadi A, Chandra P, Al Hajr M, Al Muhannadi H: **Physical Activity Profile of Adolescence in the State of Qatar**. *International Journal of Nutrition and Growth* 2015, **1**(1):1-7.

60. *Donnelly TT, Al Suwaidi J, Al Enazi NR, Idris Z, Albulushi AM, Yassin K, Rehman AM, Hassan AH: **Qatari women living with cardiovascular diseases-challenges and opportunities to engage in healthy lifestyles**. *Health Care Women Int* 2012, **33**(12):1114-1134.

61. *Duncan MJ, Al-Hazzaa HM, Al-Nakeeb Y, Al-Sobayel HI, Abahussain NA, Musaiger AO, Lyons M, Collins P, Nevill A: **Anthropometric and lifestyle characteristics of active and inactive Saudi and British adolescents**. *Am J Hum Biol* 2014, **26**(5):635-642.

62. *El Bcheraoui C, Memish ZA, Tuffaha M, Daoud F, Robinson M, Jaber S, Mikhitarian S, Al Saeedi M, AlMazroa MA, Mokdad AH *et al*: **Hypertension and its associated risk factors in the kingdom of saudi arabia, 2013: a national survey**. *Int J Hypertens* 2014, **2014**:564679.

63. *El-Aty MA, Mabry R, Morsi M, Al-Lawati J, Al-Riyami A, El-Sayed M: **Metabolic Syndrome and Its Components: Secondary analysis of the World Health Survey, Oman**. *Sultan Qaboos University medical journal* 2014, **14**(4):e460-467.

64. *El-Ghazali S, Ibrahim JM, Kanari BM, Ismail NA: **The relationship between lifestyle and body mass index among university students in Kuwait**. *Egyptian Journal of Community Medicine* 2010, **28**(1).

65. *Farghaly NF, Ghazali BM, Al-Wabel HM, Sadek AA, Abbag FI: **Life style and nutrition and their impact on health of Saudi school students in Abha, Southwestern region of Saudi Arabia**. *Saudi Med J* 2007, **28**(3):415-421.

66. *Faris MA, Epuru S, Al-Shimmari S, Al-Shimmari E: **Alarming High Levels of Energy Drinks Consumption among School Children in Hail, Northern of Saudi Arabia**. *International Journal of Child Health and Nutrition* 2015, **4**(1):1-13.

67. *Gawwad ES: **Stages of change in physical activity, self efficacy and decisional balance among saudi university students**. *J Family Community Med* 2008, **15**(3):107-115.

68. *Gharib NM, Rasheed P: **Obesity Among Bahraini Children and Adolescents: Prevalence And Associated Factors**. *Journal of the Bahrain Medical Society* 2008, **20**(3).

69. *Grant N, Gibbs T, Naseeb TA, Al-Garf A: **Medical students as family-health advocates: Arabian Gulf University experience**. *Med Teach* 2007, **29**(5):e117-121.

70. *Hashim R, Al-Ali K: **Health of dentists in United Arab Emirates**. *Int Dent J* 2013, **63**(1):26-29.

71. *Hegazy AM, Salama BM, Elgaml AM, Alzyat AR: **Association of low back pain with vitamin D deficiency and other common risk factors: A hospital based case-control study**. *European Journal of Preventive Medicine* 2015, **3**(1):1-5.

72. *Kerkadi A, Abo-Elnaga N, Ibrahim W: **Prevalence of overweight and associated risk factors among primary female school children in Al-Ain city United Arab Emirates**. *Emirates Journal of Food and Agriculture* 2005, **17**(1).

73. *Khalaf A, Ekblom O, Kowalski J, Berggren V, Westergren A, Al-Hazzaa H: **Female university students' physical activity levels and associated factors--a cross-sectional study in southwestern Saudi Arabia**. *Int J Environ Res Public Health* 2013, **10**(8):3502-3517.

74. Kilani H, Al-Hazzaa H, Waly MI, Musaiger A: **Lifestyle Habits: Diet, physical activity and sleep duration among Omani adolescents**. *Sultan Qaboos Univ Med J* 2013, **13**(4):510-519.

75. *Kneffel Z, Goebel R, Alkhatib A: **Car-diovascular risk factors and their re-sponses to a 10 weeks training pro-gram in young Qatari adults**. *Obes Res Open J* 2015, **2**(2):57-63.

76. *Koura MR, Al-Dabal BK, Rasheed P, Al-Sowielem LS, Makki SM: **Prehypertension among young adult females in Dammam, Saudi Arabia**. *East Mediterr Health J* 2013, **19**(10):899-900.

77. *Mabry RM, Winkler EA, Reeves MM, Eakin EG, Owen N: **Associations of physical activity and sitting time with the metabolic syndrome among Omani adults**. *Obesity (Silver Spring)* 2012, **20**(11):2290-2295.

78. *Mabry RM, Winkler EA, Reeves MM, Eakin EG, Owen N: **Correlates of Omani adults' physical inactivity and sitting time**. *Public Health Nutr* 2012, **16**(1):65-72.

79. *Mabry RM, Al-Busaidi ZQ, Reeves MM, Owen N, Eakin EG: **Addressing physical inactivity in Omani adults: perceptions of public health managers**. *Public Health Nutr* 2013:1-8.

80. *Mahfouz AA, Abdelmoneim I, Khan MY, Daffalla AA, Diab MM, Al-Gelban KS, Moussa H: **Obesity and related behaviors among adolescent school boys in Abha City, Southwestern Saudi Arabia**. *J Trop Pediatr* 2008, **54**(2):120-124.

81. *Mahfouz AA, Shatoor AS, Khan MY, Daffalla AA, Mostafa OA, Hassanein MA: **Nutrition, physical activity, and gender risks for adolescent obesity in Southwestern Saudi Arabia**. *Saudi J Gastroenterol* 2011, **17**(5):318-322.

82. *Memish ZA, El Bcheraoui C, Tuffaha M, Robinson M, Daoud F, Jaber S, Mikhitarian S, Al Saeedi M, AlMazroa MA, Mokdad AH *et al*: **Obesity and associated factors--Kingdom of Saudi Arabia, 2013**. *Prev Chronic Dis* 2014, **11**:E174.

83. *Midhet FM, Sharaf FK: **Impact of health education on lifestyles in central Saudi Arabia**. *Saudi Med J* 2011, **32**(1):71-76.

84. *Moradi-Lakeh M, El Bcheraoui C, Tuffaha M, Daoud F, Al Saeedi M, Basulaiman M, Memish ZA, AlMazroa MA, Al Rabeeah AA, Mokdad AH: **Self-Rated Health Among Saudi Adults: Findings from a National Survey, 2013**. *J Community Health* 2015, **40**(5):920-926.

85. *Muhairi SJ, Mehairi AE, Khouri AA, Naqbi MM, Maskari FA, Al Kaabi J, Al Dhaheri AS, Nagelkerke N, Shah SM: **Vitamin D deficiency among healthy adolescents in Al Ain, United Arab Emirates**. *BMC Public Health* 2013, **13**:33.

86. *Musaiger AO, Al-Kandari FI, Al-Mannai M, Al-Faraj AM, Bouriki FA, Shehab FS, Al-Dabous LA, Al-Qalaf WB: **Perceived barriers to weight maintenance among university students in Kuwait: the role of gender and obesity**. *Environ Health Prev Med* 2014.

87. *Musaiger AO, Zagzoog N: **Dietary and lifestyle habits among adolescent girls in Saudi Arabia: A comparison between private and government schools**. *Nutrition & Food Science* 2013, **43**(6):605-610.

88. *Platat C, Jarrar A: **Reliability and validity of a physical activity questionnaire in children**. *Int J Food Sci Nutr* 2011, **63**(6):637-644.

89. *Rouzi AA, Al-Sibiani SA, Al-Senani NS, Radaddi RM, Ardawi MS: **Independent predictors of all osteoporosis-related fractures among healthy Saudi postmenopausal women: the CEOR Study**. *Bone* 2011, **50**(3):713-722.

90. *Sadiya A, Abdi S, Abusnana S: **Lifestyle Intervention for Weight Loss: a group-based program for Emiratis in Ajman, United Arab Emirates**. *Diabetes Metab Syndr Obes* 2016, **9**:101-108.

91. *Salman RA, Al-Rubeaan KA: **Incidence and risk factors of hypertension among Saudi type 2 diabetes adult patients: an 11-year prospective randomized study**. *J Diabetes Complications* 2009, **23**(2):95-101.

92. *Serour M, Alqhenaei H, Al-Saqabi S, Mustafa AR, Ben-Nakhi A: **Cultural factors and patients' adherence to lifestyle measures**. *Br J Gen Pract* 2007, **57**(537):291-295.

93. *Shah SM, Loney T, Sheek-Hussein M, El Sadig M, Al Dhaheri S, El Barazi I, Al Marzouqi L, Aw TC, Ali R: **Hypertension prevalence, awareness, treatment, and control, in male South Asian immigrants in the United Arab Emirates: a cross-sectional study**. *BMC Cardiovasc Disord* 2015, **15**:30.

94. *Sharaf F: **Impact of health education on compliance among patients of chronic diseases in Al Qassim, Saudi Arabia**. *Int J Health Sci (Qassim)* 2011, **4**(2):139-148.

95. Sulaiman N, Hamdan A, Al-Bedri DA-LM, Young D: **Diabetes knowledge and attitudes towards prevention and health promotion: qualitative study in Sharjah, United Arab Emirates**. *International Journal of Food Safety, Nutrition and Public Health* 2009, **2**(1):78-88.

96. *Taha AZ: **Self-reported knowledge and pattern of physical activity among school students in Al Khobar, Saudi Arabia**. *East Mediterr Health J* 2008, **14**(2):344-355.

97. *Tomar RH, Hashim MH, Al-Qahtani MH: **Effects of a 12-week aerobic training on glycemic control in type 2 diabetes mellitus male patients**. *Saudi Med J* 2013, **34**(7):757-759.

98. *Tuffaha M, El Bcheraoui C, Daoud F, Al Hussaini HA, Alamri F, Al Saeedi M, Basulaiman M, Memish ZA, AlMazroa MA, Al Rabeeah AA *et al*: **Deficiencies Under Plenty of Sun: Vitamin D Status among Adults in the Kingdom of Saudi Arabia, 2013**. *N Am J Med Sci* 2015, **7**(10):467-475.

99. *Yousef S, Eapen V, Zoubeidi T, Mabrouk A: **Behavioral Correlation with Television Viewing and Videogame Use among Children in the United Arab Emirates**. *Int J Psychiatry Clin Pract* 2013.

100. *Youssef RM, Al Shafie K, Al-Mukhaini M, Al-Balushi H: **Physical activity and perceived barriers among high-school students in Muscat, Oman**. *East Mediterr Health J* 2013, **19**(9):759-768.
